# Time Series Analysis of the Microbiota of Children Suffering From Acute Infectious Diarrhea and Their Recovery After Treatment

**DOI:** 10.3389/fmicb.2018.01230

**Published:** 2018-06-12

**Authors:** Ener C. Dinleyici, Daniel Martínez-Martínez, Ates Kara, Adem Karbuz, Nazan Dalgic, Ozge Metin, Ahmet S. Yazar, Sirin Guven, Zafer Kurugol, Ozden Turel, Mehmet Kucukkoc, Olcay Yasa, Makbule Eren, Metehan Ozen, Jose Manuel Martí, Carlos P. Garay, Yvan Vandenplas, Andrés Moya

**Affiliations:** ^1^Department of Pediatrics, Faculty of Medicine, Eskisehir Osmangazi University, Eskisehir, Turkey; ^2^Institute for Integrative Systems Biology, Catedrático José Beltrán, Valencia, Spain; ^3^Pediatric Infectious Disease Unit, Faculty of Medicine, Hacettepe University, Ankara, Turkey; ^4^Department of Pediatrics, Okmeydani Education and Research Hospital, Istanbul, Turkey; ^5^Division of Pediatric Infectious Diseases, Sisli Etfal Training and Research Hospital, Istanbul, Turkey; ^6^Division of Pediatric Infectious Diseases, Konya Training and Research Hospital, Konya, Turkey; ^7^Department of Pediatrics, Umraniye Education and Research Hospital, Istanbul, Turkey; ^8^Department of Pediatrics, Faculty of Medicine, Ege University, Izmir, Turkey; ^9^Department of Pediatric Infectious Disease Unit, Faculty of Medicine, Bezmialem Vakif University, Istanbul, Turkey; ^10^Department of Pediatrics, Goztepe Training and Research Hospital, SB Istanbul Medeniyet University, Istanbul, Turkey; ^11^Department of Pediatrics, Acibadem University Faculty of Medicine, Istanbul, Turkey; ^12^Department of Pediatrics, UZ Brussel, Vrije Universiteit Brussel, Brussels, Belgium; ^13^Area de Genómica y Salud, Fundación para el Fomento de la Investigación Sanitaria y Biomédica de la Comunidad Valenciana (FISABIO-Salud Pública), Valencia, Spain; ^14^CIBER en Epidemiología y Salud Pública (CIBEResp), Madrid, Spain

**Keywords:** microbiota, temporal analysis, rotavirus, systems biology, acute infectious diarrhea

## Abstract

Gut microbiota is closely related to acute infectious diarrhea, one of the leading causes of mortality and morbidity in children worldwide. Understanding the dynamics of the recovery from this disease is of clinical interest. This work aims to correlate the dynamics of gut microbiota with the evolution of children who were suffering from acute infectious diarrhea caused by a rotavirus, and their recovery after the administration of a probiotic, *Saccharomyces boulardii* CNCM I-745. The experiment involved 10 children with acute infectious diarrhea caused by a rotavirus, and six healthy children, all aged between 3 and 4 years. The children who suffered the rotavirus infection received *S. boulardii* CNCM I-745 twice daily for the first 5 days of the experiment. Fecal samples were collected from each participant at 0, 3, 5, 10, and 30 days after probiotic administration. Microbial composition was characterized by 16S rRNA gene sequencing. Alpha and beta diversity were calculated, along with dynamical analysis based on Taylor's law to assess the temporal stability of the microbiota. All children infected with the rotavirus stopped having diarrhea at day 3 after the intervention. We observed low alpha diversities in the first 5 days (*p*-value < 0.05, Wilcoxon test), larger at 10 and 30 days after probiotic treatment. Canonical correspondence analysis (CCA) showed differences in the gut microbiota of healthy children and of those who suffered from acute diarrhea in the first days (*p*-value < 0.05, ADONIS test), but not in the last days of the experiment. Temporal variability was larger in children infected with the rotavirus than in healthy ones. In particular, *Gammaproteobacteria* class was found to be abundant in children with acute diarrhea. We identified the microbiota transition from a diseased state to a healthy one with time, whose characterization may lead to relevant clinical data. This work highlights the importance of using time series for the study of dysbiosis related to diarrhea.

## 1. Introduction

The microbiota is intimately related with the health status of the host. In previous years, this complex community of microorganisms has been studied in depth, with special emphasis on the human gut microbiota because of its main role in a myriad of diseases (Moya and Ferrer, 2016). Moreover, the essential role of the gut microbiota in central processes, such as nutrition, immunity, and physiology, has been established. The composition of the human gut microbiota changes because of several factors, in which geography and age are of great importance (Yatsunenko et al., 2012), among other external perturbations, such as antibiotic use (Jernberg et al., 2007; Dethlefsen and Relman, 2011) and diets (David et al., 2014). Alterations in this bacterial composition influence human health through different kinds of interactions between the host and microbes (Foster et al., 2017). Intestinal communities have been demonstrated to be stable over time in healthy adults, but the behavior of the microbiota becomes noisy in perturbed conditions (Martí et al., 2017).

Acute diarrhea continues to be a leading cause of morbidity, hospitalization, and mortality worldwide (Tate et al., 2016). The rotavirus genus is the most common cause of gastroenteritis in children, and recent studies have shown that infections can cause changes in the composition of intestinal microbiota (Zhang et al., 2009). The rotavirus can infect intestinal epithelium villi cells and lead to watery diarrhea, resulting in intestinal dysbiosis that destroys the microbial barrier and, in the end, worsens the diarrhea and leads to fatal consequences in some cases (Ramig, 2004). The main method of therapy for all individuals with dehydration caused by diarrhea is the use of an oral rehydration solution (ORS). Probiotics have been proposed as a complementary therapy in the treatment of acute diarrhea; they reduce the duration and severity of diarrhea, as well as the duration of hospitalization (Guarino et al., 2014). In 2014, the European Society for Pediatric Gastroenterology, Hepatology, and Nutrition and the European Society of Paediatric Infectious Diseases published their current recommendations for children with acute gastroenteritis (AGE) (Guarino et al., 2014). They highlighted that active treatment with probiotics, in addition to ORS, is effective in reducing the duration and intensity of gastroenteritis symptoms. *Lactobacillus rhamnosus* GG, *Saccharomyces boulardii* and *Lactobacillus reuteri* DSM 17938 are the most common probiotics studied and used in children with AGE (Dinleyici et al., 2012; Dinleyici and Vandenplas, 2014; Guarino et al., 2014). Several randomized and controlled studies on *S. boulardii* have been conducted, and in-depth meta-analyses are now available on the use of this microorganism in the treatment of acute diarrhea with viral, bacterial, and protozoan causes in both developing and developed countries (Dinleyici et al., 2012, 2014). *S. boulardii* can reduce the duration of diarrhea by approximately 1 day, shorten the initial phase of watery stools, and decrease the length of hospital stay (Dinleyici et al., 2012). Our recent study in a clinical trial with probiotics in acute gastroenteritis (PROBAGE Study), which is a multicenter, randomized, prospective, controlled, single-blind clinical trial, showed that *S. boulardii* CNCM I-745 reduced the duration of diarrhea in both in- and out-patient settings, as well as reduced the length of emergency care unit and hospital stay among children below 5 years of age with acute infectious diarrhea (Dinleyici et al., 2015). The probiotic effect started to be observed at 48 h of intervention, and major beneficial effects were seen at 72 h. A recent meta-analysis showed that in 9 out of 11 clinical trials, *S. boulardii* was beneficial in children with acute infectious diarrhea, as it significantly reduced the duration of their acute infectious diarrhea by approximately 24 h compared with the controls (Dinleyici et al., 2012).

The changes in microbial composition related to diseases are known by the concept of dysbiosis. It has been observed that gut microbiota is related to a significant number of well-defined diseases such as irritable bowel syndrome (IBS) (Durbán et al., 2013), Crohn's disease (Morgan et al., 2012), obesity (Turnbaugh et al., 2009; Ferrer et al., 2013), colorectal cancer (Peters et al., 2016), or HIV (Vázquez-Castellanos et al., 2014; Serrano-Villar et al., 2016) to cite some examples. Acute gastroenteritis can be considered as a related disease to other as IBS or Crohn's regarding dysbiosis. It has been observed that people affected by IBS had an increased abundance of *Firmicutes*, especially of *Ruminococcaceae* spp. and *Clostridium* cluster XIVa, with the reduction of *Bacteroides*, (Jeffery et al., 2011). In the case of Crohn's disease, it has been observed that *Faecalibacterium prausnitzii* and *Escherichia coli*, in particular, were decreased in patients suffering from CD (Pascal et al., 2017). Concerning dysbiosis caused by rotavirus and norovirus infection in children, it has been observed a decrease of microbial diversity compared to children without infection, and differential abundances of *Prevotella, Staphylococcus* or *Atopobium* genera (Chen et al., 2017). At any rate, whether dysbiosis is cause or effect of diseases is a matter of discussion, with a non-trivial answer most of the times (Gareau et al., 2010).

In this study, called the Fecal microbiota Analysis of Children receiving probiotics due to acute Infectious Diarrhea (FACID), we aimed to explore the temporal evolution of the microbiota of children with acute infectious diarrhea caused by a rotavirus. We examined how the microbiota of these children improved after the administration of *S. boulardii* CNCM I-745 plus ORS during the first 5 days of the experiment, compared with the microbiota of a healthy group of children of the same age, over a period of 30 days.

## 2. Materials and methods

### 2.1. Sample selection

FACID Study aims to evaluate the description and behavior of the fecal microbiota of children caused by acute infectious diarrhea due to a rotavirus infection. We enrolled a total of 16 children, 6 of them with a healthy condition, and the other 10 suffering from acute infectious diarrhea. All children were between 36 and 48 months old; this age range was selected because the microbiota is finally stabilized during this stage (Rodríguez et al., 2015), and so that the differences caused by age disparity between groups are reduced. Median age of children was 36 months. On admission, children were clinically examined and the fever and degree of dehydration were recorded. Two out of them had fever at admission and resolved in 24 hours of hospitalization. Seven children have mild to moderate dehydratation. The duration of diarrhea was defined as the time in hours from admission until cessation of diarrhea, which in turn was defined as the first normal stool according to Bristol score (a score under five is described as normalization of stool). In rotavirus diarrhea group, all children had no diarrhea at the 3rd day of intervention. The duration of diarrhea varied from 24 to 72 h (median 72 h). Specific clinical data about dehydration, fever and diarrhea duration in children with rotavirus diarrhea is included in Supplementary Table 1. To eliminate the potential biases associated with the intestinal microbiota during infancy, we selected children who were normally delivered, breastfed for at least 6 months, and have not received antibiotic therapy since birth. From each child enrolled, we collected samples at five different time points, namely, at 0, 3, 5, 10, and 30 days, for a total of 80 different samples. The sample at day 0 was taken before probiotic treatment in the group of children with infectious diarrhea. All children belonging to the infectious diarrhea group received oral lyophilized *S. boulardii* CNCM I-745 (250 mg twice daily, Reflor, Biocodex) for the first 5 days, specifically days 3 and 5 (day 0 was with no intervention), in addition to ORS and/or intravenous therapy. These children were diagnosed to have a rotavirus infection with a rapid immunoassay test. Rehydration and electrolyte replacement were done using hypoosmolar ORS (glucose 20 g; sodium 60 mmol/L; potassium 20 mmol/L; bicarbonate 30 mmol/L). The frequency and consistency of the stools were recorded. The duration of diarrhea was defined as the time in hours from admission until the cessation of diarrhea, which, in turn, was defined as the first normal stool according to the Bristol score (a score under five is described as a normalization of stool).

The Eskisehir Osmangazi University Local Ethics Committee from Turkey reviewed and approved the protocol. Prior to the collection of the children's stool and data, consent was given by their parents. This study was registered in *ClinicalTrials.gov* with the accession code *NCT01927094*. All samples were stored at -80°C until the DNA extraction. All the fecal samples were transferred into sterile Falcon tubes and stored at -80°C until further steps. The fecal samples were weighed to extract the total DNA using the QIAamp DNA Stool Mini Kit (Qiagen, Hilden, Germany), in accordance with the manufacturer's instructions. The extracted DNA was shipped under cold shipment to FISABIO in Valencia, Spain for further analysis. More information about the participants is available in Supplementary Table 2.

### 2.2. 16S rRNA gene sequencing and read processing

A region of the 16S rRNA gene (V4) was selected to be studied. Sequencing was performed on the Illumina MiSeq platform according to the manufacturer's specifications. Sequencing was conducted at the Sequencing and Bioinformatics Service of FISABIO Foundation. 16S rRNA gene reads were quality filtered with PRINSEQ, lite version (Schmieder and Edwards, 2011). Reads with a low score (<20) and short read lengths (<50 nucleotides) were removed. Chimeric sequence filtering and taxonomic assignment of the 16S rRNA sequences were performed with the ChimeraSlayer algorithm and open-reference workflow, respectively, in QIIME (v 1.8) (Caporaso et al., 2010), with default parameter values. Operational taxonomic units (OTUs) were clustered at 97% similarity with the Uclust algorithm. The most representative sequences for each OTU were compared with those in the Greengenes database (representative sequences aligned at 97% similarity, August 2013). Only those annotations that had more than 0.6 in their bootstrap confidence estimation values were accepted for further analyses.

### 2.3. Diversity and functional analyses

Alpha diversity estimators were computed with R programming language using the vegan package (v. 2.4-3) (Oksanen et al., 2017). We applied two filters on the OTU table that resulted from QIIME: any OTU that had less than 80 counts in total was deleted, and any OTU that had a count of 0 in more than 65 samples was excluded from the analysis. Shannon entropy and Pielou evenness were computed. Then, the true diversity index based on Shannon entropy was also computed with the exponentiation of the Shannon values. This index has been proven as a true (and linear) estimator of the diversity of a population (Jost, 2007). While having twice of Shannon entropy in one sample with respect to another does not imply that we have double of diversity because of the non-linear nature of this index, the Shannon true diversity index is linear and relies on the term of effective species. Differences in alpha diversities were studied by performing permutations over the Wilcoxon signed-rank test with a significance level of 0.05, and it was calculated using the coin package in R (Hothorn et al., 2008). In the case of multiple comparisons, we applied false discovery rate correction to avoid type I errors.

For beta diversity, canonical correspondence analysis (CCA) was performed by using the vegan package in R programming language. We applied the function ADONIS within the vegan package, a multivariate ANOVA based on dissimilarity tests, to assess the variables that were statistically significant in the separation of the samples in two dimensions. We used the linear discriminant analysis (LDA) effect size (LEfSe) algorithm from the Galaxy software package of Huttenhower lab to identify the specific taxa that served as the biomarkers for patients with probiotic treatment and the controls (Segata et al., 2011). LEfSe first determines the significant differences in taxa composition between groups by using non-parametric factorial Kruskal-Wallis sum-rank test. Then, LEfSe uses LDA to estimate the effect size of each differentially abundant feature. We fixed a cut-off alpha value of < 0.05 for the Kruskal-Wallis test, and for the bacterial taxa that had significant differences between samples, we fixed a threshold of 4.0 in the logarithmic LDA score to consider it a discriminative feature. We split the dataset into two subsets: the first one containing the days 0, 3, and 5, and the second one grouping days 10 and 30. This separation was made to have, on one hand, the time points of the probiotic treatment and, on the other hand, the time points without the probiotic treatment. We performed both CCA and LEfSe analysis on the resulting subsets.

For functional content prediction, we used the PICRUSt software from Huttenhower laboratory (Langille et al., 2013), in which we feed the software with the taxonomical information of the 16S ribosomal gene, and then it extracts the metabolic information of the community. We used default parameters and the KEGG database for inference (Kanehisa, 2000; Kanehisa et al., 2015, 2016). We extracted the information at levels 2 and 3 in KEGG hierarchies, as we have more specificity when we increase the level of hierarchy. To assess the differences in function abundances, we applied the Kruskal-Wallist test, with the α value fixed at 0.01.

### 2.4. Temporal analysis and stability

Temporal analysis was conducted according to Martí et al. (2017) to measure the stability, over time, of all the individuals. We use the Taylor's Law, an ecological empirical law which is a power law dependence between mean relative abundance *x*_*i*_ and dispersion σ_*i*_ ($\sigma_{i}=V\cdot x_{i}^{\beta }$). In this model, we have two free parameters, *V* and β. Parameter *V* is a direct measure of the amplitude of temporal fluctuations and, thus, the most informative parameter in this case. β is the scaling factor, and it is always less than 1, implying that the most abundant taxa are less susceptible to perturbations than the less-abundant ones. The importance of this method is that it captures relevant temporal information about the microbial communities, which can be related, in the end, to the health status of the host. Taylor's parameters were computed and standardized in the same manner as in the original work (in standard deviation units), with those samples of children with acute infectious diarrhea compared with the ones of the healthy individuals in the study. We also computed the rank stability of all samples, which shows time differences per participant. For this purpose, we calculated the rank stability index (RSI), which shows the temporal stability of a certain taxon, by considering its rank in the total population. RSI is calculated as 1 minus the quotient of the true rank hops, divided by the maximum possible rank hops, all powered to an arbitrary *p* power:

(1) $$\begin{array}{r} RSI={\left(1-\frac{truerankhops}{possiblerankhops}\right)}^{p} \end{array}$$

We also calculated the *rank variability*, which is the absolute difference between each taxon rank and the overall rank, as well as the *differences variability*, which is the absolute difference between each taxon rank at a given time and the value it had in the previous time step, averaged over all taxa present.

### 2.5. Data accession number

The microbiome data from this study are available at NCBI SRA Database under Bioproject ID PRJNA416445. Information about the participants of the study can be found Additional File 1.

## Results

### Bacterial composition and diversity

**Figure 1:**
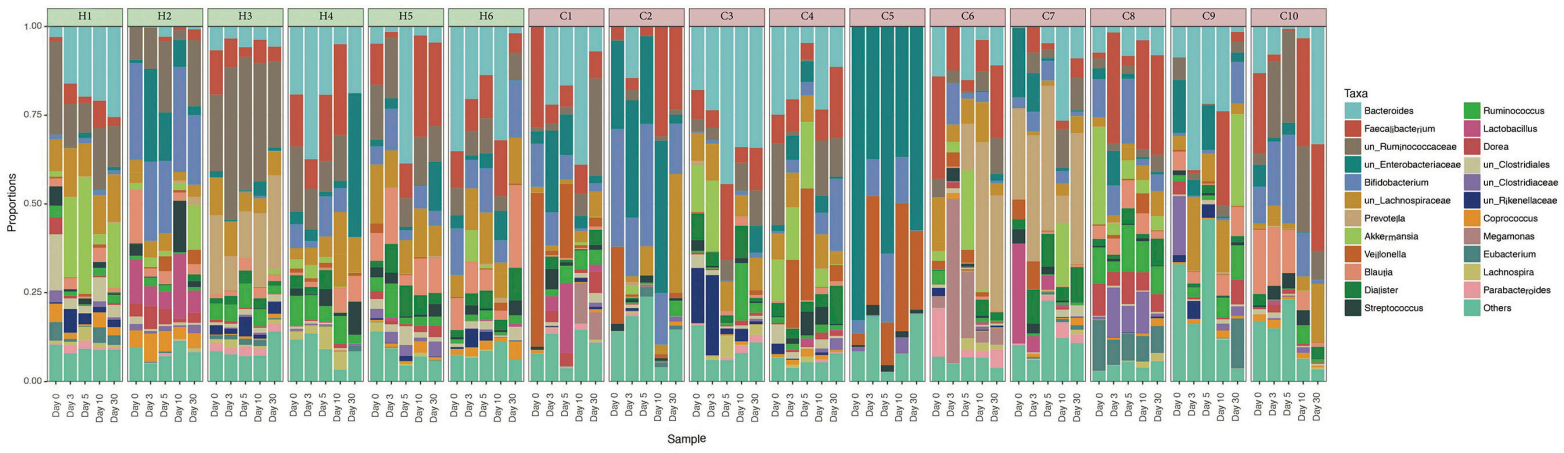
Relative abundances of gut microbiota in every individual. Taxonomic relative abundances at genus level of each individual, separated per day of sampling. Healthy children (H1–H6) are marked in green, and cases (C1–C10) are marked in red. Only the 25 most abundant OTUs overall were represented. The less abundant OTUs were joined into the *Others* group.

A total of 3,664,535 sequences of 16S rRNA gene amplicons from 80 samples passed the quality filters and were assigned to a taxonomy with QIIME. Each sample had an average number of 45,806 sequences with an standard deviation of 12,598 sequences. The 25 most abundant taxa are illustrated in Figure 1, and the remaining taxa were joined into a single group named as *Other*. The most abundant genus is *Bacteroides* (13.14%), followed by *Faecalibacterium* (9.6%) and two unclassified genera from the *Ruminococcaceae* (9.35%) and *Enterobacteriaceae* (8.02%) families respectively. The abundance profile of the children has a high heterogeneity. We can see that the *Bacteroides* genus is most abundant in patients C1, H1, H4, or H5, whereas *Faecalibacterium* is in higher proportions in patients C2, C8, C10, or H5, depending on the day we made the observation. C5 is dominated by the unclassified genera from *Enterobacteriaceae*, as this is the sample with the clearest dominance of one OTU over the others, in comparison with the rest of the participants. We can also observe that the *Other* group fluctuates at a higher degree in the unhealthy group. From the analysis of patient variables from children with rotavirus infection (available in Supplementary Table 1), we did not find any statistical difference neither grouping by dehydration nor by diarrhea duration (*p*-value >0.05, *t*-test).

**Figure 2:**
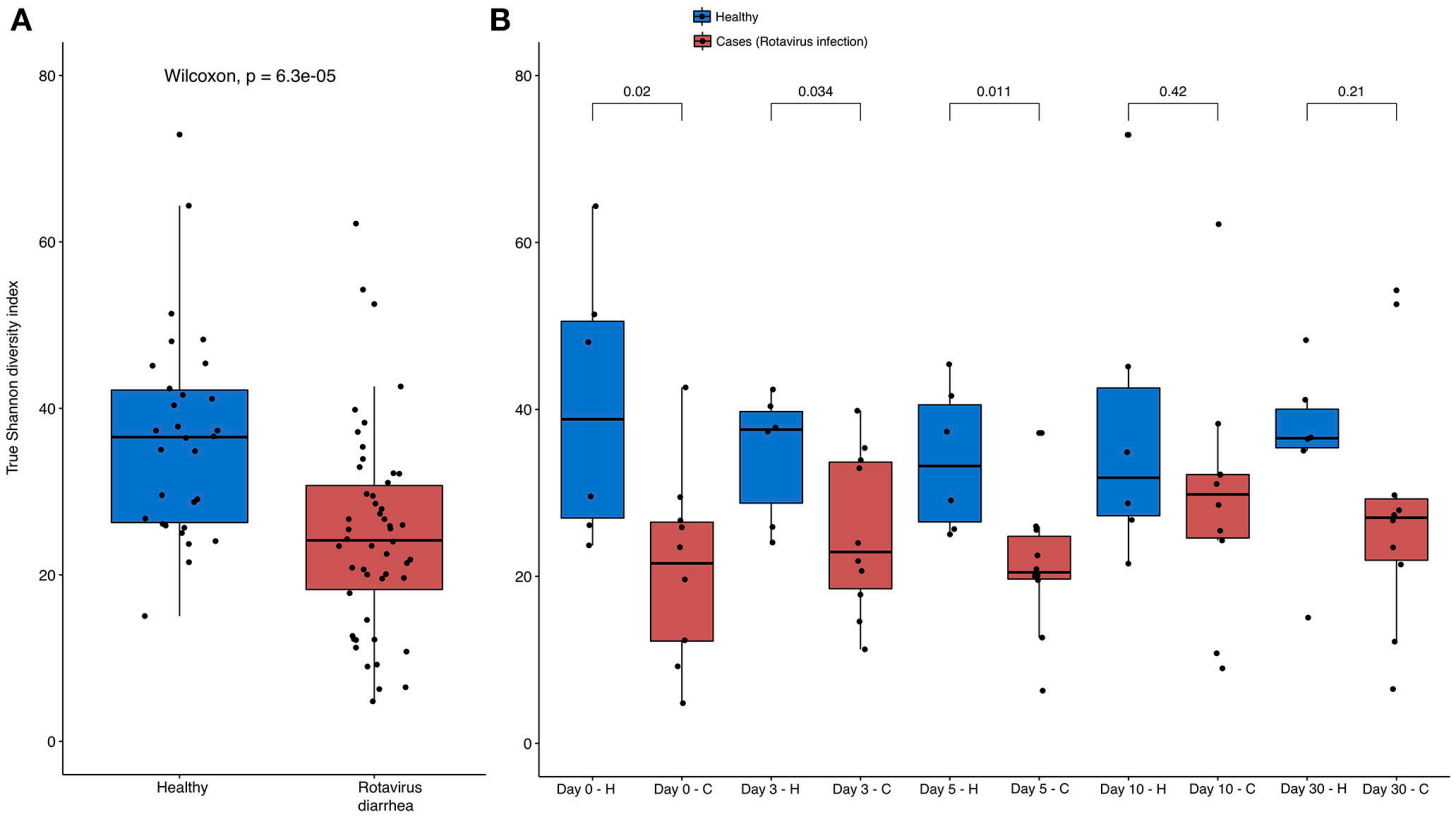
Shannon diversity index per health status and per day Boxplots showing the Shannon diversity index between **(A)** both health status with all times together; and **(B)**, separated by time of sampling. Color blue represents healthy children, and red color represents children with acute diarrhea. *P*-value of Wilcoxon test is showed in the upper part of each comparison in both parts of the Figure, and the different health states are represented by H and C letters in part B for healthy and cases respectively. All data points were represented using the function jitter in R.

The analysis of alpha diversity with the Shannon diversity index, which compared both health states and considered all times together, reveals that healthy and non-healthy children are statistically different in the Wilcoxon test (*p*-value = 6.3e-05), with a higher diversity in the former group than in the latter (see Figure 2). When we compare the differences between states, but per time, we find statistical differences at day 0 (*p*-value = 0.02), day 3 (*p*-value = 0.034) and day 5 (*p*-value = 0.011), the period that corresponds to the treatment. On the contrary, we did not find any statistical significance at day 10 (*p*-value = 0.42) nor day 30 (*p*-value = 0.21), which corresponds to the period after the treatment, when all children had healthy conditions. This increase in diversity is correlated with the health status recovery of the children. All the tests were also applied to Pielou's Evenness index (Supplementary Figure 1), and the results were almost exactly the same except that no significant difference was observed between the healthy and sick states at day 3 (*p*-value = 0.093).

**Figure 3:**
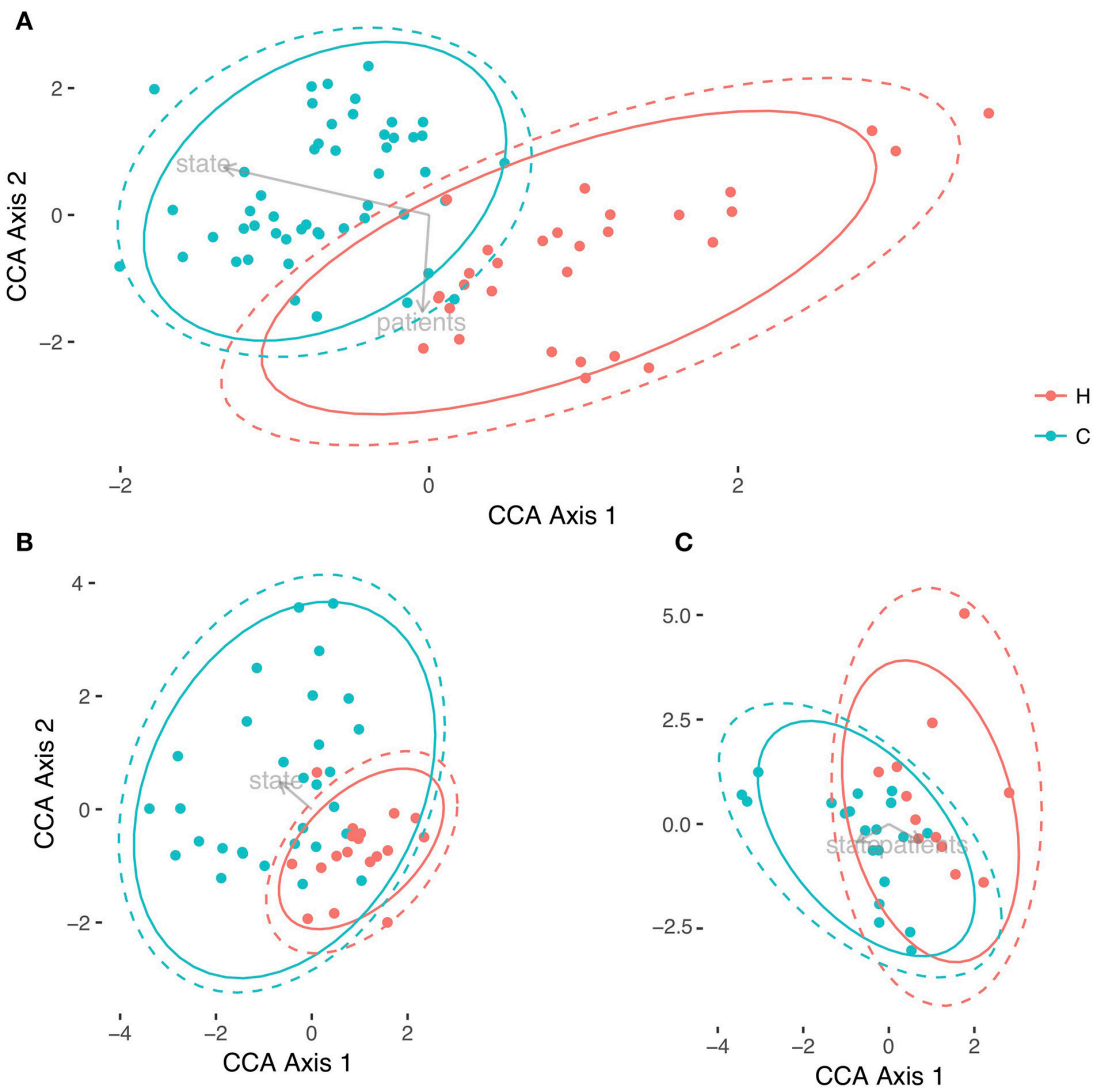
Comparison of microbiotas between healthy children and children with acute diarrhea. Three different representation of samples with Canonical Correspondence Analysis. In **(A)** is the global differences between both health status, Healthy (H, in red), and Cases (C, in green) for all times; in **(B)** we represented the CCA for the individuals from time 0 to 5, and in **(C)** it is represented the CCA of samples belonging to days 10 and 30. Both health status were circled in their respective colors with no error, and with 95% of Confidence Level in all Figure parts.

We performed a Canonical Correspondence Analysis (CCA) to the OTU community matrix, with an ADONIS test to assess the variables that were separating the samples with statistical significance. Not only we found statistical differences between the individuals of the experiment (*p*-value = 0.001), but also between the health status of the host (*p*-value = 0.009). Both variables separate the samples in two directions (see Figure 3). As can be observed, a clear separation exists between the community matrix belonging to healthy children compared with the children suffering from the rotavirus infection. By contrast, more overlapping is present between individuals when this variable is observed in the CCA (see Supplementary Figure 2). This result points to a differential composition in the communities depending on the health state. To determine whether this separation between health status was consistent with the results of alpha diversity, we applied CCA and the ADONIS test to the dataset split in two different subsets: the first one corresponding to days 0 through 5, and the second one corresponding to days 10 and 30. Running the ADONIS test on these two subsets revealed statistical differences in the status of the first group (*p*-value = 0.019), but not in the second group (*p*-value = 0.289). In the first case, the region of the healthy children is more constrained and centered than that of the children with acute diarrhea; in the second case, both regions overlap their area (see Figures 3B,C). These results reinforce the results obtained by alpha diversity, highlighting that we have differences in the first three time points, but not in the last two.

### Taxonomic and functional enrichment

**Figure 4:**
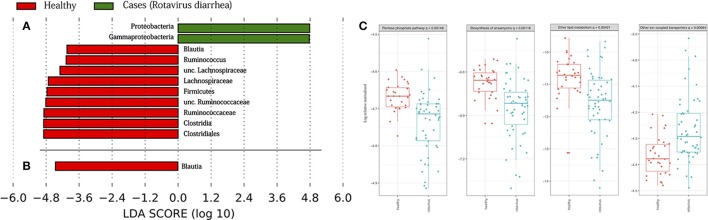
Taxonomic biomarkers and functions enriched. Linear discriminative analysis (LDA) effect size (LEfSe) analysis between the healthy children (in red) and case children (in green), in **(A)** from days 0 to 5, and in **(B)** from days 10 to 30. LDA scores (log 10) for the most prevalent taxa in controls are represented on the negative side, whereas LDA-positive scores indicate enriched taxa in cases. In **(C)** are represented the KEGG (Kyoto Encyclopedia of Genes and Genomes) pathways at level 3 of hierarchy validated by Kruskal-Wallis test.

To determine which taxa were responsible for the differences between host status, we performed the LEfSe analysis test in the two aforementioned subsets (days 0–5, and days 10–30), which is represented in Figures 4A,B. We found that in the case of the subset from days 0, 3, and 5, there was an enrichment of bacteria belonging to phylum *Proteobacteria*, specifically the *Gammaproteobacteria* class. On the other hand, only phylum *Firmicutes* was abundant in the healthy children, in which we can find genera, such as *Blautia, Ruminococcus*, or other bacteria belonging to the *Lachnospiraceae* and *Ruminococcaceae* families. By contrast, the analysis of the second subset showed that only the genus *Blautia* was abundant in the healthy children. The results from PICRUSt revealed a higher stability in functions, in general, with respect to the variability seen at the taxonomic level (see Supplementary Figure 3). The Kruskal-Wallis test showed the statistically significant differences in the metabolism of other amino acids at level 2 in KEGG hierarchy in the comparison of both health states. The same test was also significant for level 3 in KEGG, as we observed differences between four groups: the pentose phosphate pathway, the biosynthesis of ansamycins, ether lipid metabolism, and other ion-coupled transporters (see Figure 4C).

### Dynamic analysis of the microbiota

**Figure 5:**
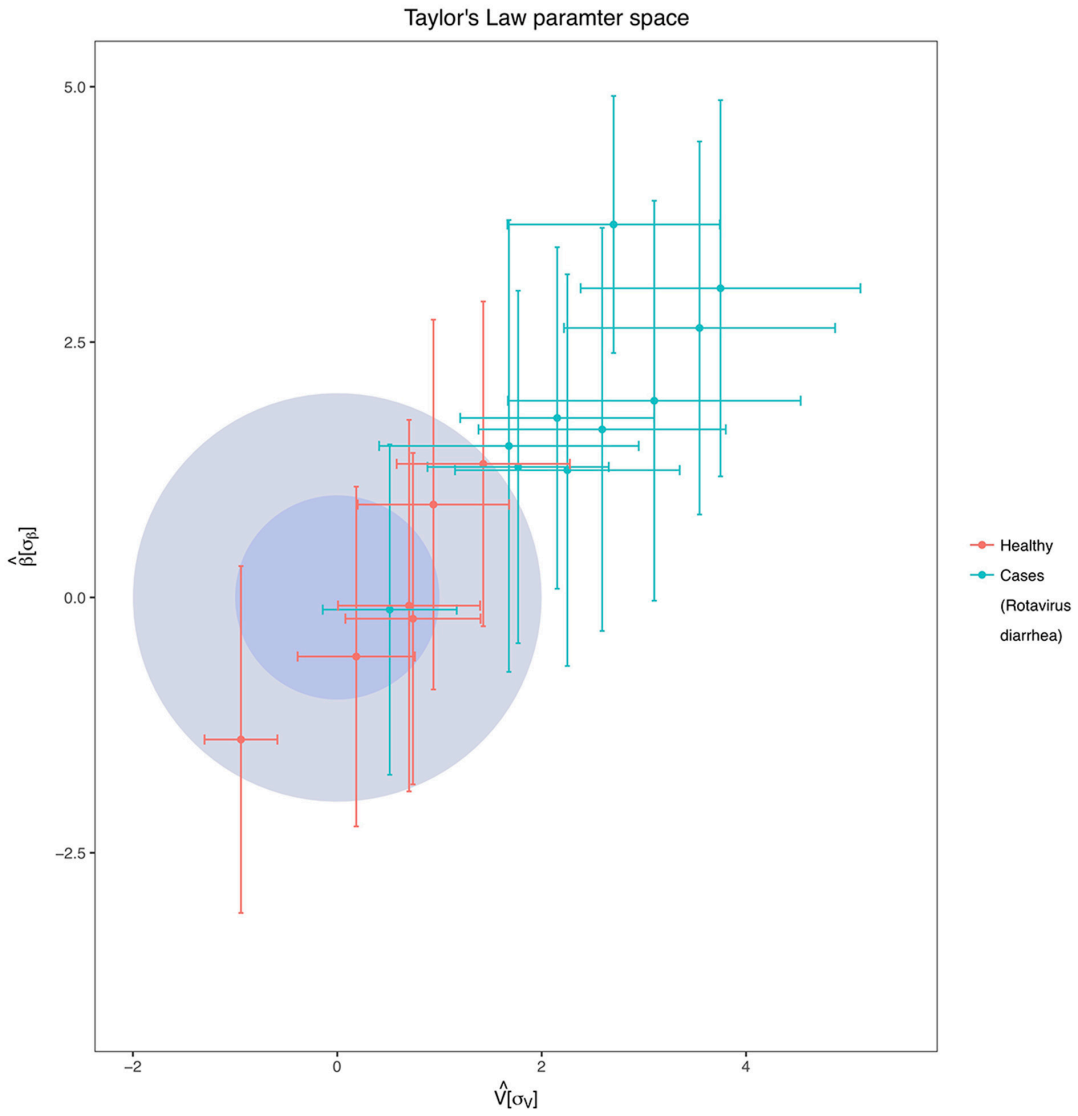
Taylor's Law Parameter Space. The inner darker-blue circle corresponds to the 68% CL region of healthy children in the Taylor's parameter space, while the bigger light-blue circle delimits the 98% CL region. In the Figure are represented both the healthy children (red points with error bars) and children with acute diarrhea (green points with error bars). Taylor's parameters were standardized as mentioned in Material and Methods, and they have standard deviation units.

A temporal analysis was conducted to evaluate the temporal stability of the individuals over time. Of the two free parameters of the Taylor's Law fit, β was always less than 1 in all individuals, and we found pronounced differences between the values of the variability *V* (see Supplementary Table 3). Figure 5 shows the standardization of the parameters concerning the healthy group. We can observe that all children suffering from acute diarrhea (except child C8) are categorized under the healthy zone, meaning they have a higher temporal variability than the healthy individuals of the study. The lower stability of children with rotavirus was in agreement with the other results from this section, as their microbiomes have been suffering changes in time in respect to the healthy children, which have been stable through the time of sampling. All the children who are outside of the *healthy zone* are, at least, two σ units distant from the center in terms of *V*.

**Figure 6:**
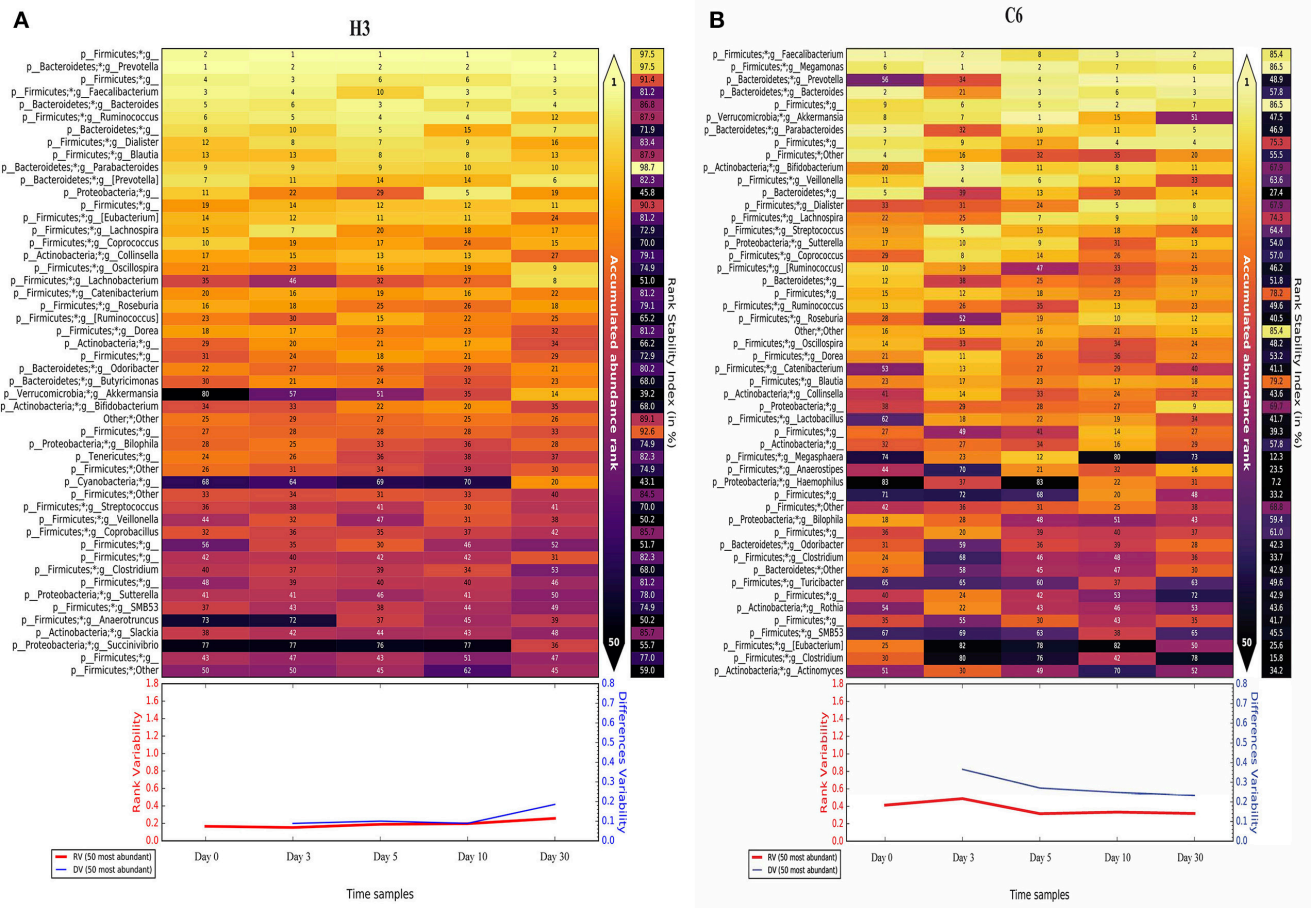
Rank Stability Matrix. Rank Stability Matrix for the most variable subject (H3, **A**), and the least variable subject (C6, **B)**. In both plots are represented the 50 most abundant genera of each case, and the numbers inside each cell represents the ranking of that specific genus at that specific time point. The color inside each cell ranges from light-yellow for the rank 1, to black, representing very low ranks. At the right in each case it is shown the Rank Stability Index, and below them it is represented the Rank Variability (in red) and the Differences Variability (in blue).

We also analyzed the rank stability of the individuals. We represent the two samples with the least and the greatest variability in the temporal analysis, which are H3 (a healthy child) and C6 (a non-healthy child), respectively (see Figure 6). We can see that the RSI has lower values in the non-healthy individual than in the healthy one. H3 has 33 taxa out of a total of 50, with an RSI above 70%, whereas C6 has only 8 taxa above that threshold. Some taxa also appear to be very stable despite their low proportions in the last 20 positions. An example is the case of genus *Actinomyces*, which appears to be very stable at low proportions (around rank 40) in four of the six healthy children. There are other genera of interest, such as *Collinsela*, which also seems to be very stable in healthy children, but it alternates cases where it has low proportions with others in which is highly abundant in the affected children. Lastly, the case of *Blautia* is interesting; it is very stable and has high proportions in all healthy children, but it is absent in some of the children infected with the rotavirus and is found in low proportions in other cases. This finding is consistent with the LeFSe results. The general tendency of these less-abundant taxa is to belong to the phylum *Firmicutes*. Finally, as seen in Figure 6, we can observe that both the rank variability and the differences variability are significantly lower in H3 than in C6, an indicator that is also present in all healthy children compared with the non-healthy group (all individuals are shown in Supplementary File 1).

## 3. Discussion

The heterogeneity observed in the abundance profile of every individual in this study is expected because of the inherent diversity that has been proven to exist in the human gut microbiota (Lozupone et al., 2012). Several factors can cause this heterogeneity, such as host genetics (Goodrich et al., 2014), microbial assembly (Costello et al., 2012; Franzenburg et al., 2013) and type of birth (Rodríguez et al., 2015), although the latter is controversial (Falony et al., 2016). Nevertheless, the most abundant taxa belonged to *Firmicutes* and *Bacteroidetes*, which are the dominant phyla in adult microbiota, in accordance with the literature (Turnbaugh et al., 2009; Claesson et al., 2011). Specifically, the finding that *Bacteroides* is one of the most abundant genera is also consistent with literature results (The et al., 2017). One interesting observation that can be made is the higher proportion and variability of the *Others* group in the children with acute diarrhea. This finding reveals two interesting features of the experiment: first, these unhealthy microbiotas showed a higher instability than the healthy subset, and second, they had a higher representation of some taxa that were rather a minority in healthy individuals.

The results of the alpha diversity analysis showed a transition from a low-diversity to a high-diversity condition in the children with acute diarrhea. The relationship between low diversity and diseases in the gut microbiota has been extensively demonstrated (Lozupone et al., 2012), and this seems to be in agreement with the results of our experiment. Overall, the low alpha diversity at diarrhea stages and its recovery to a normal diversity is in accordance with the findings of previous works (Pop et al., 2014; Subramanian et al., 2014; Chen et al., 2017), but it is important to remark that some of these studies were developed in low income countries and malnourished infants. Moreover, the beta diversity analysis with the CCA and the ADONIS test revealed that we had different communities at the individual and condition levels. This means that not only were the microbiotas of the unhealthy children less diverse, but they had a different configuration of microbes, compared with those of the healthy children. In agreement with the alpha diversity results, these differences seemed to appear only in the first time points of the experiment during the treatment. At days 10 and 30, the statistical differences disappeared, which means that the communities in both groups of children were similar. Differences found at the individual level are also in agreement with the heterogeneity observed in Figure 1.

From the temporal analysis, a very good distinction between both groups of children was found in terms of population dynamics. Temporal stability has been proved to be related to the health status of the host (Martí et al., 2017), and in this study, we observed that almost every child infected with the rotavirus had a lower temporal stability than the healthy children. It is important to note that a higher temporal variability is, to a certain extent, caused by the transition from the diseased state to a healthy one. This transition is marked by changes in the bacteria that live in the gut, along with their relative abundance. Nevertheless, a high temporal variability during the diarrhea process is expected because of different reasons, such as the rapid succession of microbes resulting from continuous and rapid fecal movement. The reason why participant C8 remained in the stable part in Figure 5 is intriguing, but it is worth noting that C8 is the child that had the fastest recovery compared to the other patients. He was the only affected child in which diarrhea disappeared 24 h later. With this quick recovery, C8 microbiome would have been stabilized earlier than the other affected children. The rank stability plots presented some interesting trends, as well, because of the presence of some groups of bacterial genera that are stable even at a low abundance. The case of *Actinomyces* is particularly intriguing, as it seems to inhabit these low-abundance regimes in a very stable manner (Martí et al., 2017) in the healthy children. Some species from this genus are observed to be in higher proportions in other cases of diarrhea (The et al., 2017). Because of their opportunistic nature, limiting these species to live in low abundance could be beneficial for the host.

The microbial communities of children suffering from diarrhea are less mature than those of healthy children (Subramanian et al., 2014). It could be that some of the differences we see might be due for this effect, along to the aforementioned microbial assembly processes that occurs in the first stages of the human life. Suffering diseases as the acute infectious diarrhea could delay the maturation to the adult microbiota (David et al., 2015). This high variability in non-healthy children agrees with the other results of this study, as it seems that their microbiotas improve from a perturbed state to a healthy one with time. This is one of the most important points of this experiment, as it proves the power of time series analysis in microbiota, especially in cases in which a disease is being examined.

As we stated, the microbiota has a different configuration of microbes in both health states, and the differences between them could be of clinical interest. LEfSe analysis showed a clear differentiation in terms of the phyla enriched, with a high amount of *Proteobacteria* observed during the first few days in the children suffering from acute diarrhea. This result agrees with those of extant literature, as an enrichment of bacteria, such as *Escherichia coli* (a *Gammaproteobacteria*), in children suffering diarrhea was found (Kotloff et al., 2013; Pop et al., 2014; Chen et al., 2017). On the contrary, *Firmicutes* phylum is usually lowered in children with diarrhea, so an enrichment of this group was expected in healthy children (Monira et al., 2013). Compared to other dysbiosis is interesting to notice that patients suffering from irritable bowel syndrome had an elevated abundance of *Firmicutes*, including *Ruminococcus* spp., members of genera *Clostridium* and *Staphylococcus* (Jeffery et al., 2011), what seems to be contrary to our results. However, it has also been observed that there might be a direct relationship between having an acute gastroenteritis, and the start of exacerbation of irritable bowel diseases (Rodríguez et al., 2006). Nevertheless, irritable bowel diseases are complex conditions, and most surely the microbiota is not the unique factor that can start or enhance patient's symptoms. Interestingly, we did not see much differences regarding functions enriched. The most intriguing case is the pathway involved in the biosynthesis of ansamycins, a secondary bacterial metabolite that has antimicrobial properties against a broad range of bacteria (Binda et al., 1971). Some analogs of ansamycins interact with Hsp90, which helps inhibit the severity of the infection by rotavirus (Dutta et al., 2009). Although being only an *in vitro* work, this could be of future interest when the study is expanded to new directions.

In summary, this is the first study characterizing the microbiota of children with acute infectious diarrhea in time, caused by a rotavirus, where we have shown that microbiota composition seems to correlate with clinical improvement. Children stopped experiencing most of the symptoms 3 days after probiotic administration, and alongside, microbiota recovered both its diversity and healthy configuration within this period. Therefore, gut microbiota seems to play an essential role in diarrhea-related processes. Whether gut microbiota is an active actor or a mere spectator should be addressed in future studies with other experimental designs, and an in-depth study of the functions that carry its different components.

## Author contributions

Conception and design: ED and AM. Enrollment, patient follow-up, data recording: AdK, ND, OM, AY, SG, ZK, OT, MK, OY, MO, ME. Data analysis: DM-M. Interpretation of data: DM-M, AM, ED. Draft writing: DM-M, AM, ED. Draft Revision: ED, DM-M, AtK, JM, CG, YV, AM. Approval of the submitted version: All authors.

### Conflict of interest statement

This study has been totally supported and funded by the Turkish Pediatric Probiotic Prebiotic and Microbiota Society. ED has been a consultant and member of Biocodex Advisory Board, and serves as a consultant and speaker for Biocodex and BioGaia. YV has participated as a clinical investigator, and/or advisory board member, and/or consultant, and/or speaker for Abbott Nutrition, Aspen, Biocodex, Danone, Nestle Health Science, Nestle Nutrition Institute, Nutricia, Phacobel, and United Pharmaceuticals. The remaining authors declare that the research was conducted in the absence of any commercial or financial relationships that could be construed as a potential conflict of interest. The reviewer AC and handling Editor declared their shared affiliation.

## Supplementary material

Supplementary Figure 1 Boxplots showing the Evenness diversity index between both health status with all times together; and separated by time of sampling. Color blue represents healthy children, and red color represents children with acute diarrhea.

Supplementary Figure 2 Canonical Correspondence Analysis of all individuals and times, separated by different child.

Supplementary Figure 3 Relative abundances of the KEGG pathways inferred by PICRUSt at level 2 of the hierarchy.

Supplementary Table 1 Excel file with clinical data. Dehydration, fever and diarrhea duration are the variables included.

Supplementary Table 2 Excel file with subject information of this study. It is specified the hospital where the subjects were treated, the amount of DNA extracted, and the ratios of purity.

Supplementary Table 3 Excel file which summarizes the values of Taylor's parameters *V* and β. In the file you can see information about each individual of this study: number of OTUs, times, mean of absolute frequencies, standard deviation of absolute frequencies, weighted values for *V*, β and their errors, and weighted values of the standardization of both parameters with their respective errors.

Supplementary File 1 Rank Stability matrices for every subject enrolled in the study. In each Figure, are represented the 50 most abundant genera of each subject, and the numbers inside each cell represents the ranking of that specific genus at that specific time point. The color inside each cell ranges from light-yellow for the rank 1 to black, representing very low ranks. At the right in each case it is shown the Rank Stability Index, and below them it is represented the Rank Variability (in red) and the Differences Variability (in blue).
