# Supplementary figures and images for: Time Series Analysis of the Microbiota of Children Suffering From Acute Infectious Diarrhea and Their Recovery After Treatment
Source: Front Microbiol. 2018 Jun 12;9:1230. doi: 10.3389/fmicb.2018.01230 (PMC6005867; doi:10.3389/fmicb.2018.01230)

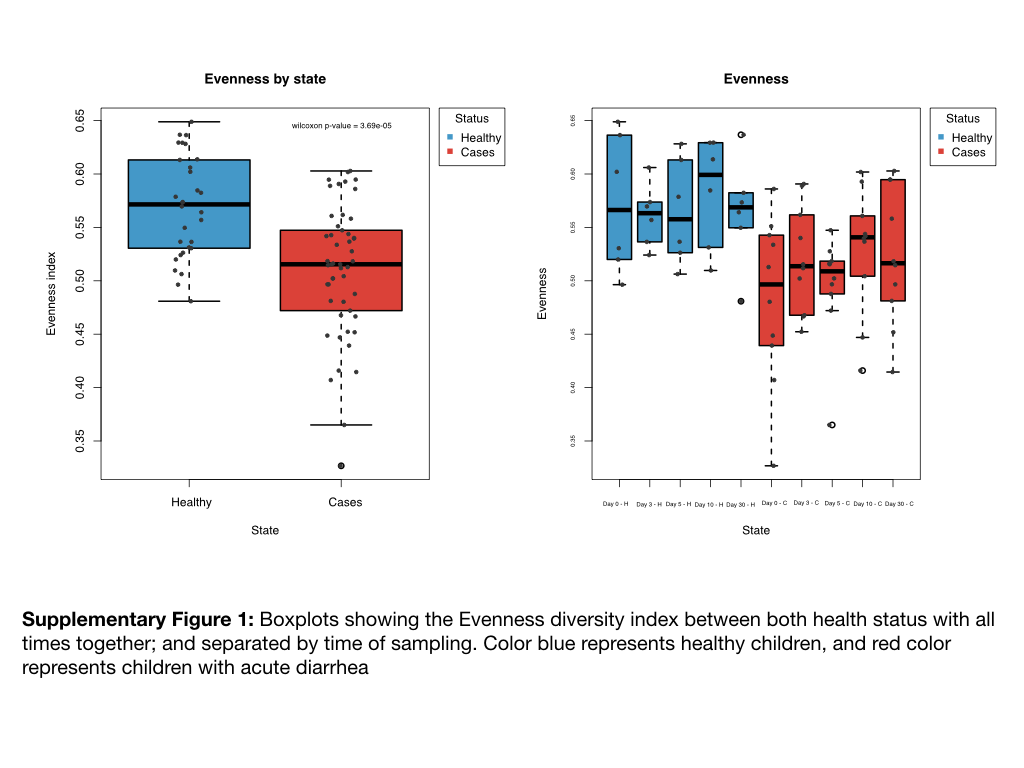

Supplement: Supplementary Figure 1 — Boxplots showing the Evenness diversity index between both health status with all times together; and separated by time of sampling. Color blue represents healthy children, and red color represents children with acute diarrhea. [file Image_1.TIFF]

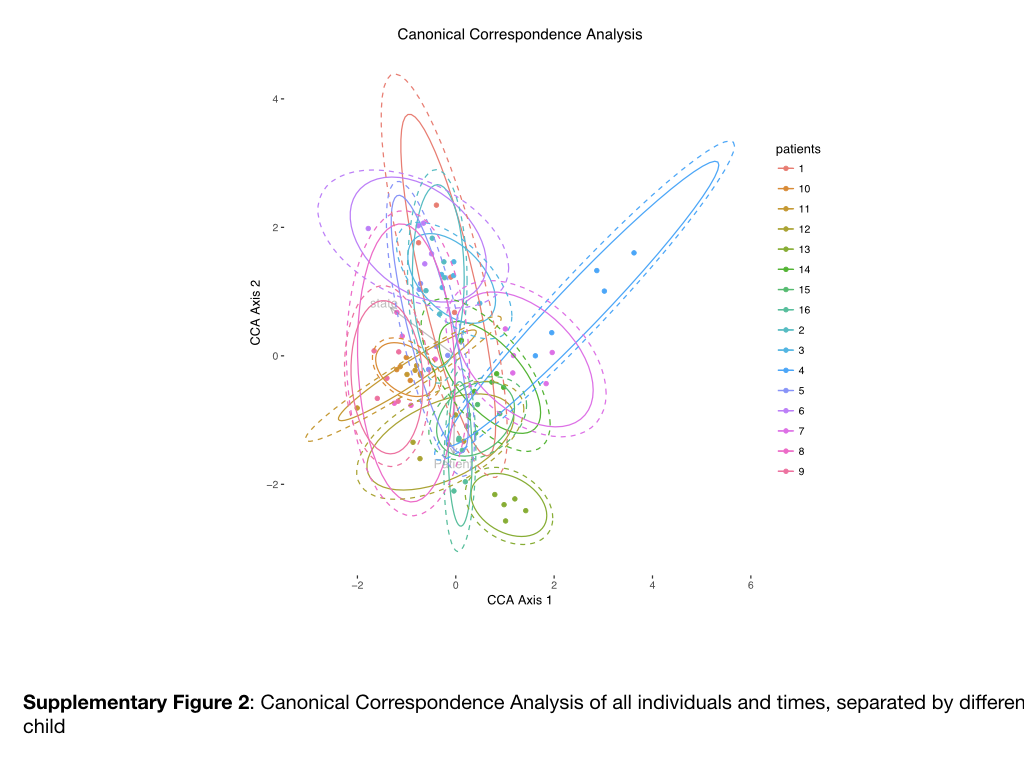

Supplement: Supplementary Figure 2 — Canonical Correspondence Analysis of all individuals and times, separated by different child. [file Image_2.TIFF]

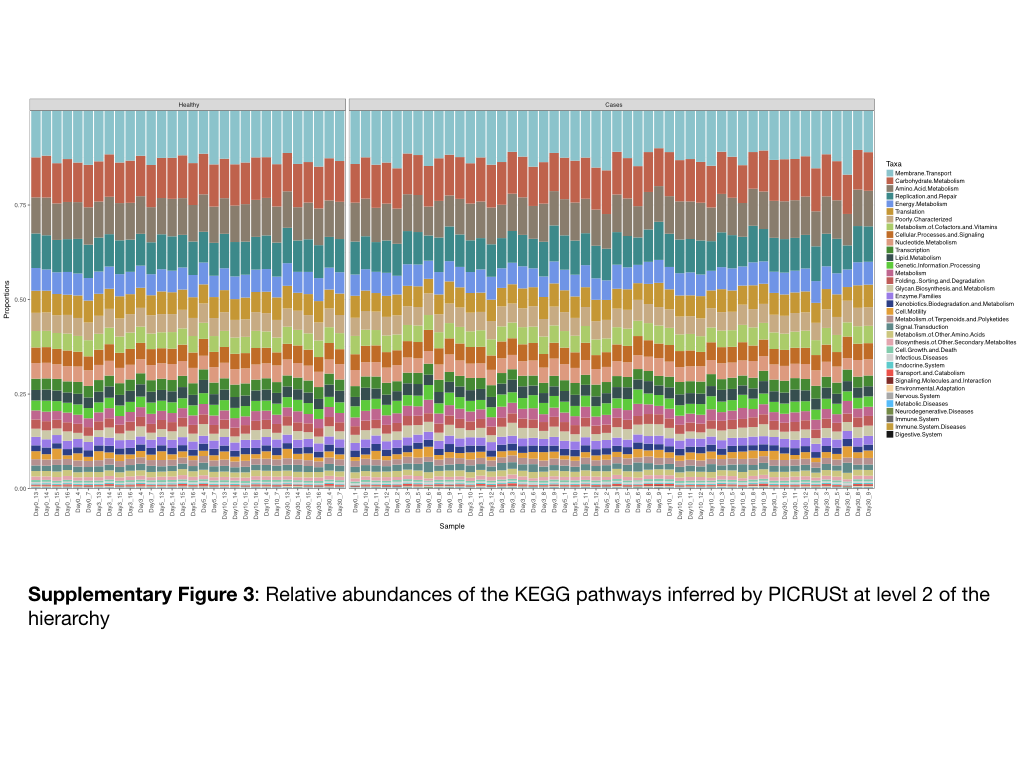

Supplement: Supplementary Figure 3 — Relative abundances of the KEGG pathways inferred by PICRUSt at level 2 of the hierarchy. [file Image_3.TIFF]
