# Supplementary material for: Time Series Analysis of the Microbiota of Children Suffering From Acute Infectious Diarrhea and Their Recovery After Treatment
Source: Front Microbiol. 2018 Jun 12;9:1230. doi: 10.3389/fmicb.2018.01230 (PMC6005867; doi:10.3389/fmicb.2018.01230)
Supplement: Supplementary File 1 — Rank Stability matrices for every subject enrolled in the study. In each Figure, are represented the 50 most abundant genera of each subject, and the numbers inside each cell represents the ranking of that specific genus at that specific time point. The color inside each cell ranges from light-yellow for the rank 1 to black, representing very low ranks. At the right in each case it is shown the Rank Stability Index, and below them it is represented the Rank Variability (in red) and the Differences Variability (in blue). [file Data_Sheet_1.pdf]

Additional File 1: Rank Stability matrices for every subject enrolled in the study. In each Figure, are represented the 50 most abundant genera of each subject, and the numbers inside each cell represents the ranking of that specific genus at that specific time point. The color inside each cell ranges from light-yellow for the rank 1 to black, representing very low ranks. At the right in each case it is shown the Rank Stability Index, and below them it is represented the Rank Variability (in red) and the Differences Variability (in blue).

H1

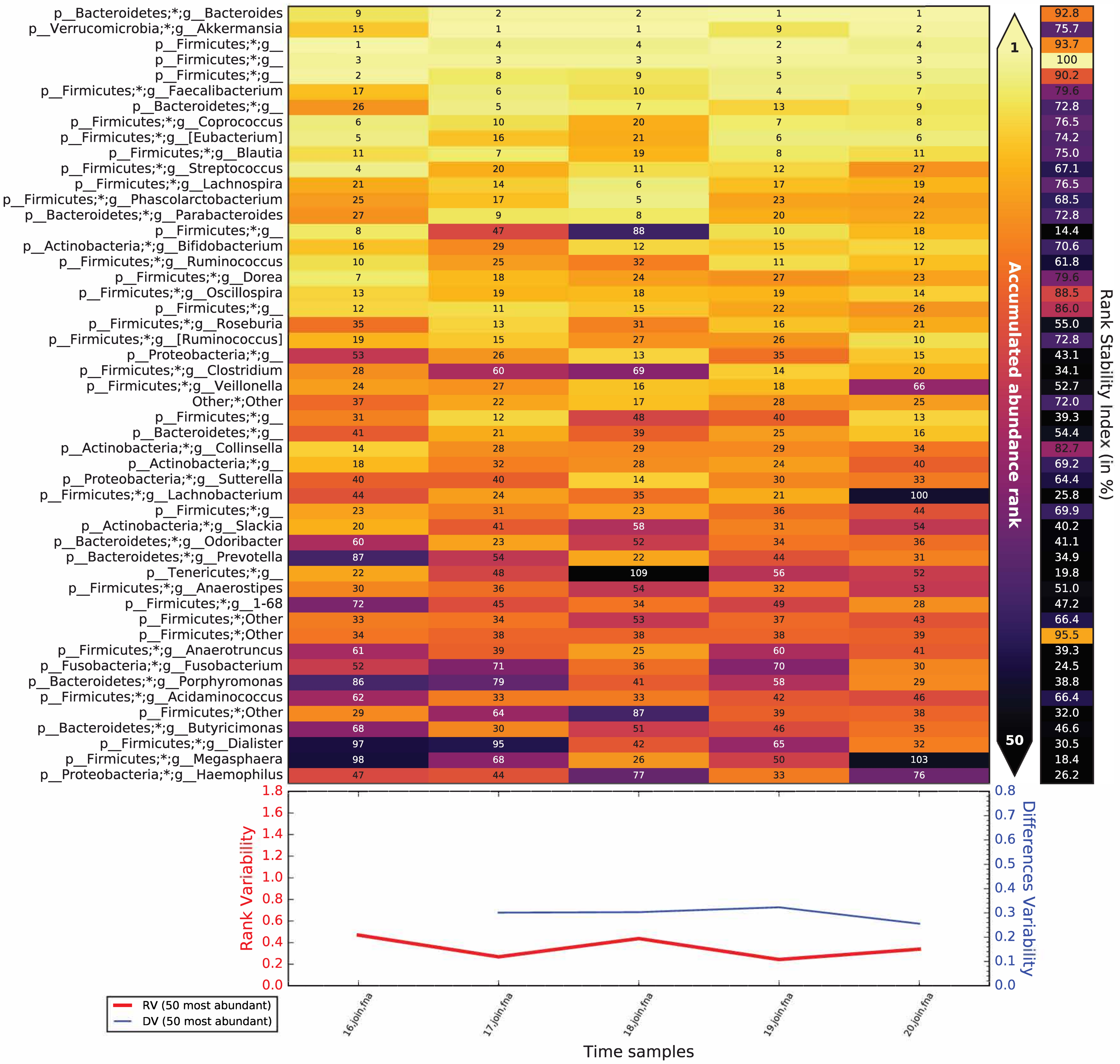
p__Bacteroidetes;*;g__Bacteroides 9 2 2 1 1 92.8
p__Verrucomicrobia;*;g__Akkermansia 15 1 1 9 2 75.7
p__Firmicutes;*;g__ 1 4 4 2 4 93.7
p__Firmicutes;*;g__ 3 3 3 3 3 100
p__Firmicutes;*;g__ 2 8 9 5 5 90.2
p__Firmicutes;*;g__Faecalibacterium 17 6 10 4 7 79.6
p__Bacteroidetes;*;g__ 26 5 7 13 9 72.8
p__Firmicutes;*;g__Coprococcus 6 10 20 7 8 76.5
p__Firmicutes;*;g__[Eubacterium] 5 16 21 6 6 74.2
p__Firmicutes;*;g__Blautia 11 7 19 8 11 75.0
p__Firmicutes;*;g__Streptococcus 4 20 11 12 27 67.1
p__Firmicutes;*;g__Lachnospira 21 14 6 17 19 76.5
p__Firmicutes;*;g__Phascolarctobacterium 25 17 5 23 24 68.5
p__Bacteroidetes;*;g__Parabacteroides 27 9 8 20 22 72.8
p__Firmicutes;*;g__ 8 47 88 10 18 14.4
p__Actinobacteria;*;g__Bifidobacterium 16 29 12 15 12 70.6
p__Firmicutes;*;g__Ruminococcus 10 25 32 11 17 61.8
p__Firmicutes;*;g__Dorea 7 18 24 27 23 79.6
p__Firmicutes;*;g__Oscillospira 13 19 18 19 14 88.5
p__Firmicutes;*;g__ 12 11 15 22 26 86.0
p__Firmicutes;*;g__Roseburia 35 13 31 16 21 55.0
p__Firmicutes;*;g__[Ruminococcus] 19 15 27 26 10 72.8
p__Proteobacteria;*;g__ 53 26 13 35 15 43.1
p__Firmicutes;*;g__Clostridium 28 60 69 14 20 34.1
p__Firmicutes;*;g__Veillonella 24 27 16 18 66 52.7
Other;*;Other 37 22 17 28 25 72.0
p__Firmicutes;*;g__ 31 12 48 40 13 39.3
p__Bacteroidetes;*;g__ 41 21 39 25 16 54.4
p__Actinobacteria;*;g__Collinsella 14 28 29 29 34 82.7
p__Actinobacteria;*;g__ 18 32 28 24 40 69.2
p__Proteobacteria;*;g__Sutterella 40 40 14 30 33 64.4
p__Firmicutes;*;g__Lachnobacterium 44 24 35 21 100 25.8
p__Firmicutes;*;g__ 23 31 23 36 44 69.9
p__Actinobacteria;*;g__Slackia 20 41 58 31 54 40.2
p__Bacteroidetes;*;g__Odoribacter 60 23 52 34 36 41.1
p__Bacteroidetes;*;g__Prevotella 87 54 22 44 31 34.9
p__Tenericutes;*;g__ 22 48 109 56 52 19.8
p__Firmicutes;*;g__Anaerostipes 30 36 54 32 53 51.0
p__Firmicutes;*;g__1-68 72 45 34 49 28 47.2
p__Firmicutes;*;Other 33 34 53 37 43 66.4
p__Firmicutes;*;Other 34 38 38 38 39 95.5
p__Firmicutes;*;g__Anaerotruncus 61 39 25 60 41 39.3
p__Fusobacteria;*;g__Fusobacterium 52 71 36 70 30 24.5
p__Bacteroidetes;*;g__Porphyromonas 86 79 41 58 29 38.8
p__Firmicutes;*;g__Acidaminococcus 62 33 33 42 46 66.4
p__Firmicutes;*;Other 29 64 87 39 38 32.0
p__Bacteroidetes;*;g__Butyricimonas 68 30 51 46 35 46.6
p__Firmicutes;*;g__Dialister 97 95 42 65 32 30.5
p__Firmicutes;*;g__Megasphaera 98 68 26 50 103 18.4
p__Proteobacteria;*;g__Haemophilus 47 44 77 33 76 26.2
1
Accumulated abundance rank
50
Rank Stability Index (in %)
Rank Variability
1.8
1.6
1.4
1.2
1.0
0.8
0.6
0.4
0.2
0.0
Differences Variability
0.8
0.7
0.6
0.5
0.4
0.3
0.2
0.1
0.0
RV (50 most abundant)
DV (50 most abundant)
16.join.fna
17.join.fna
18.join.fna
19.join.fna
20.join.fna
Time samples

H2

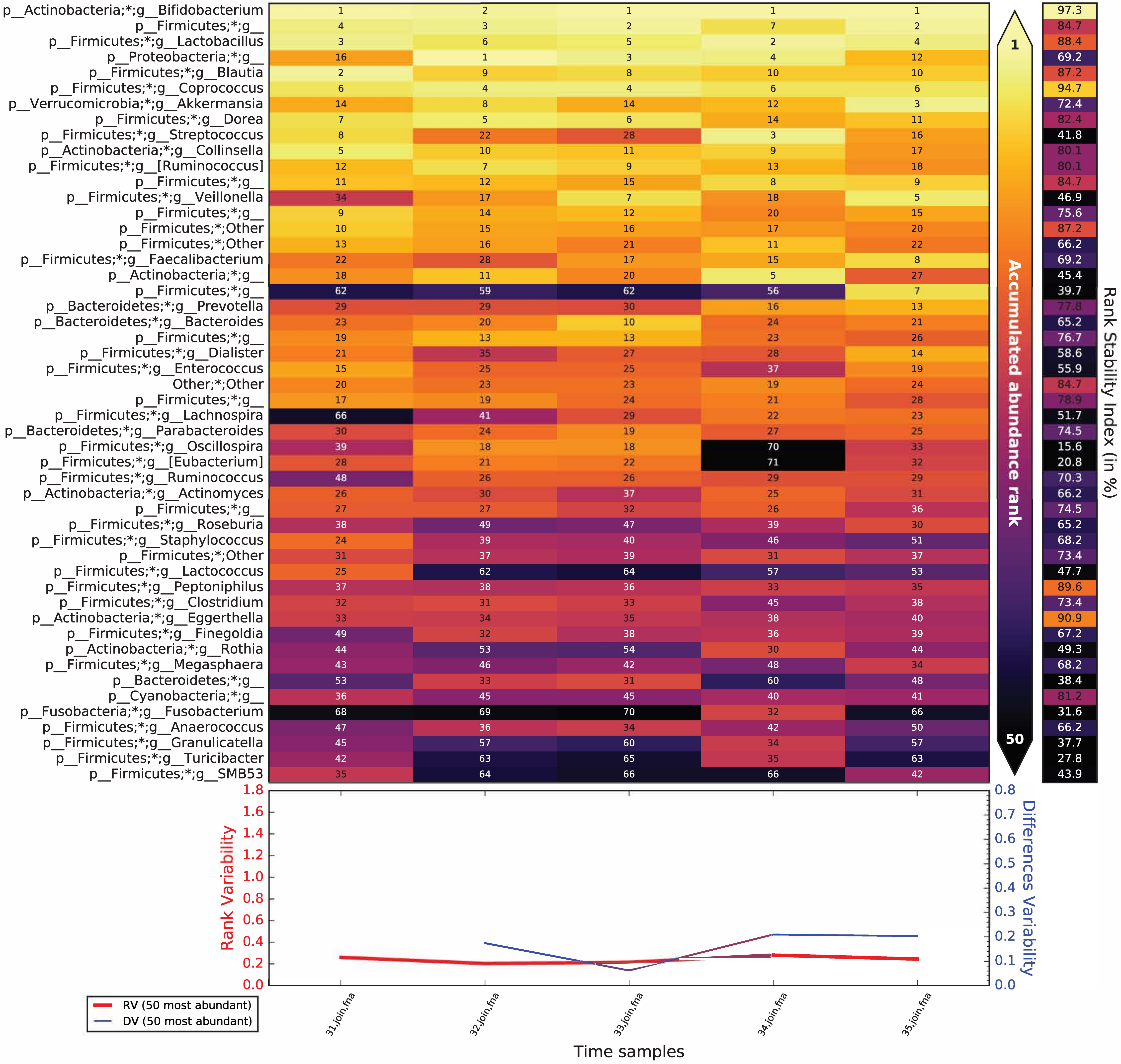

p__Actinobacteria;*;g__Bifidobacterium
p__Firmicutes;*;g__
p__Firmicutes;*;g__Lactobacillus
p__Proteobacteria;*;g__
p__Firmicutes;*;g__Blautia
p__Firmicutes;*;g__Coprococcus
p__Verrucomicrobia;*;g__Akkermansia
p__Firmicutes;*;g__Dorea
p__Firmicutes;*;g__Streptococcus
p__Actinobacteria;*;g__Collinsella
p__Firmicutes;*;g__[Ruminococcus]
p__Firmicutes;*;g__
p__Firmicutes;*;g__Veillonella
p__Firmicutes;*;g__
p__Firmicutes;*;Other
p__Firmicutes;*;Other
p__Firmicutes;*;g__Faecalibacterium
p__Actinobacteria;*;g__
p__Firmicutes;*;g__
p__Bacteroidetes;*;g__Prevotella
p__Bacteroidetes;*;g__Bacteroides
p__Firmicutes;*;g__
p__Firmicutes;*;g__Dialister
p__Firmicutes;*;g__Enterococcus
Other;*;Other
p__Firmicutes;*;g__
p__Firmicutes;*;g__Lachnospira
p__Bacteroidetes;*;g__Parabacteroides
p__Firmicutes;*;g__Oscillospira
p__Firmicutes;*;g__[Eubacterium]
p__Firmicutes;*;g__Ruminococcus
p__Actinobacteria;*;g__Actinomyces
p__Firmicutes;*;g__
p__Firmicutes;*;g__Roseburia
p__Firmicutes;*;g__Staphylococcus
p__Firmicutes;*;Other
p__Firmicutes;*;g__Lactococcus
p__Firmicutes;*;g__Peptoniphilus
p__Firmicutes;*;g__Clostridium
p__Actinobacteria;*;g__Eggerthella
p__Firmicutes;*;g__Finegoldia
p__Actinobacteria;*;g__Rothia
p__Firmicutes;*;g__Megasphaera
p__Bacteroidetes;*;g__
p__Cyanobacteria;*;g__
p__Fusobacteria;*;g__Fusobacterium
p__Firmicutes;*;g__Anaerococcus
p__Firmicutes;*;g__Granulicatella
p__Firmicutes;*;g__Turicibacter
p__Firmicutes;*;g__SMB53
Accumulated abundance rank
1
50
Rank Stability Index (in %)
97.3
84.7
88.4
69.2
87.2
94.7
72.4
82.4
41.8
80.1
80.1
84.7
46.9
75.6
87.2
66.2
69.2
45.4
39.7
77.8
65.2
76.7
58.6
55.9
84.7
78.9
51.7
74.5
15.6
20.8
70.3
66.2
74.5
65.2
68.2
73.4
47.7
89.6
73.4
90.9
67.2
49.3
68.2
38.4
81.2
31.6
66.2
37.7
27.8
43.9
Rank Variability
Differences Variability
RV (50 most abundant)
DV (50 most abundant)
31.join.fna
32.join.fna
33.join.fna
34.join.fna
35.join.fna
Time samples

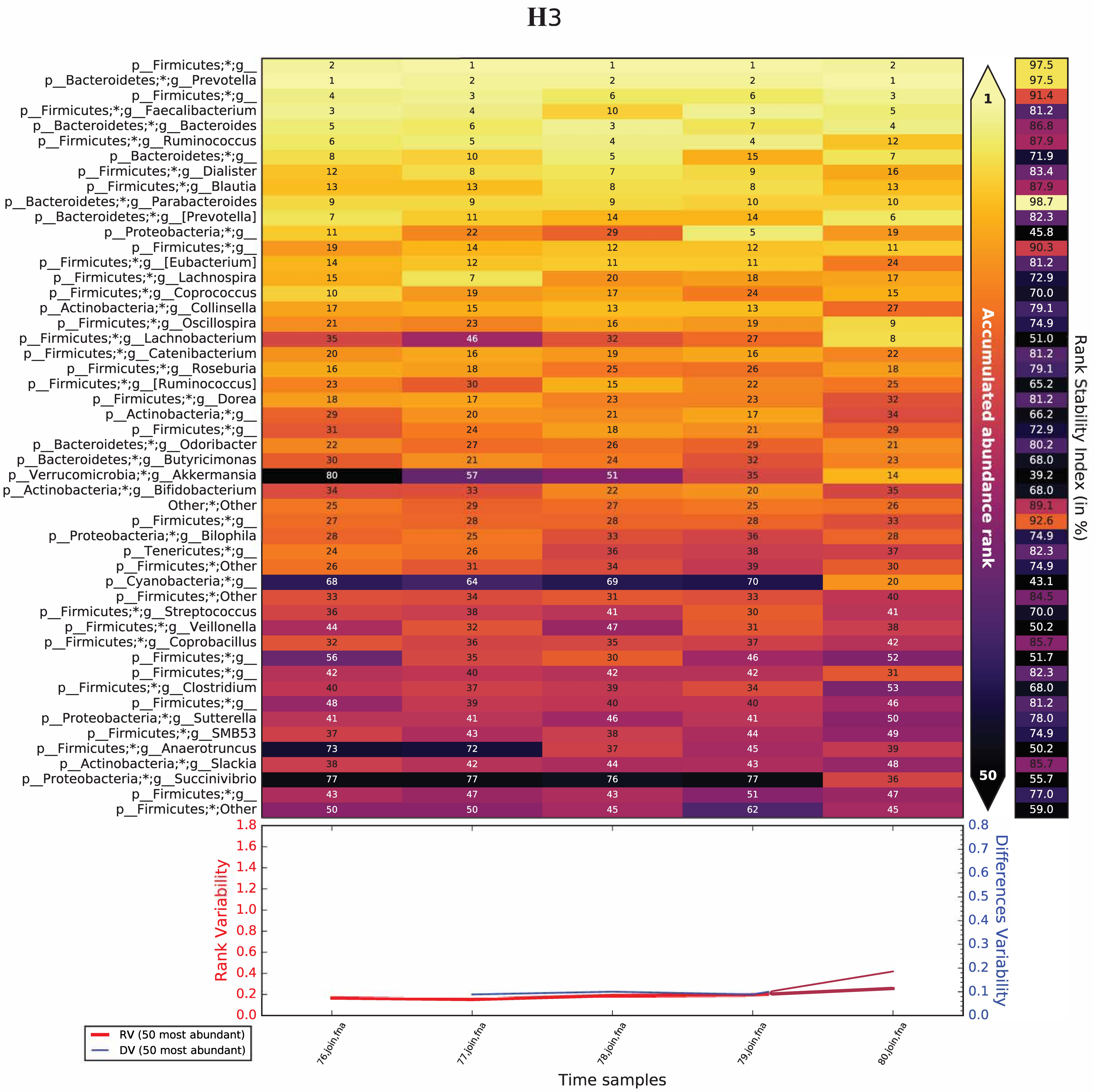
H3
p__Firmicutes;*;g__
p__Bacteroidetes;*;g__Prevotella
p__Firmicutes;*;g__
p__Firmicutes;*;g__Faecalibacterium
p__Bacteroidetes;*;g__Bacteroides
p__Firmicutes;*;g__Ruminococcus
p__Bacteroidetes;*;g__
p__Firmicutes;*;g__Dialister
p__Firmicutes;*;g__Blautia
p__Bacteroidetes;*;g__Parabacteroides
p__Bacteroidetes;*;g__[Prevotella]
p__Proteobacteria;*;g__
p__Firmicutes;*;g__
p__Firmicutes;*;g__[Eubacterium]
p__Firmicutes;*;g__Lachnospira
p__Firmicutes;*;g__Coprococcus
p__Actinobacteria;*;g__Collinsella
p__Firmicutes;*;g__Oscillospira
p__Firmicutes;*;g__Lachnobacterium
p__Firmicutes;*;g__Catenibacterium
p__Firmicutes;*;g__Roseburia
p__Firmicutes;*;g__[Ruminococcus]
p__Firmicutes;*;g__Dorea
p__Actinobacteria;*;g__
p__Firmicutes;*;g__
p__Bacteroidetes;*;g__Odoribacter
p__Bacteroidetes;*;g__Butyricimonas
p__Verrucomicrobia;*;g__Akkermansia
p__Actinobacteria;*;g__Bifidobacterium
Other;*;Other
p__Firmicutes;*;g__
p__Proteobacteria;*;g__Bilophila
p__Tenericutes;*;g__
p__Firmicutes;*;Other
p__Cyanobacteria;*;g__
p__Firmicutes;*;Other
p__Firmicutes;*;g__Streptococcus
p__Firmicutes;*;g__Veillonella
p__Firmicutes;*;g__Coprobacillus
p__Firmicutes;*;g__
p__Firmicutes;*;g__
p__Firmicutes;*;g__Clostridium
p__Firmicutes;*;g__
p__Proteobacteria;*;g__Sutterella
p__Firmicutes;*;g__SMB53
p__Firmicutes;*;g__Anaerotruncus
p__Actinobacteria;*;g__Slackia
p__Proteobacteria;*;g__Succinivibrio
p__Firmicutes;*;g__
p__Firmicutes;*;Other
Accumulated abundance rank
1
50
Rank Stability Index (in %)
97.5
97.5
91.4
81.2
86.8
87.9
71.9
83.4
87.9
98.7
82.3
45.8
90.3
81.2
72.9
70.0
79.1
74.9
51.0
81.2
79.1
65.2
81.2
66.2
72.9
80.2
68.0
39.2
68.0
89.1
92.6
74.9
82.3
74.9
43.1
84.5
70.0
50.2
85.7
51.7
82.3
68.0
81.2
78.0
74.9
50.2
85.7
55.7
77.0
59.0
Rank Variability
Differences Variability
RV (50 most abundant)
DV (50 most abundant)
76.join.fna
77.join.fna
78.join.fna
79.join.fna
80.join.fna
Time samples

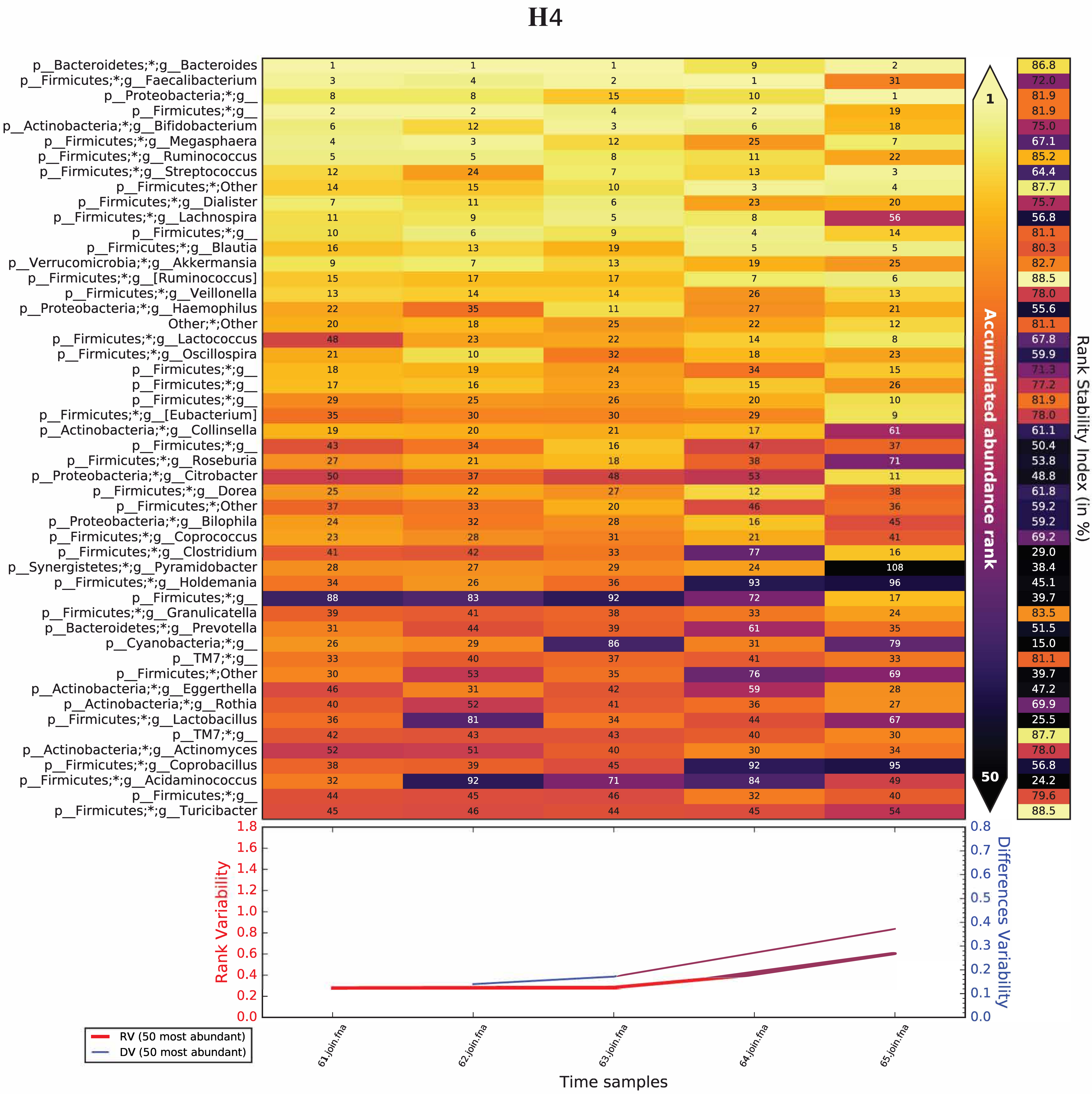
H4
p__Bacteroidetes;*;g__Bacteroides
p__Firmicutes;*;g__Faecalibacterium
p__Proteobacteria;*;g__
p__Firmicutes;*;g__
p__Actinobacteria;*;g__Bifidobacterium
p__Firmicutes;*;g__Megasphaera
p__Firmicutes;*;g__Ruminococcus
p__Firmicutes;*;g__Streptococcus
p__Firmicutes;*;Other
p__Firmicutes;*;g__Dialister
p__Firmicutes;*;g__Lachnospira
p__Firmicutes;*;g__
p__Firmicutes;*;g__Blautia
p__Verrucomicrobia;*;g__Akkermansia
p__Firmicutes;*;g__[Ruminococcus]
p__Firmicutes;*;g__Veillonella
p__Proteobacteria;*;g__Haemophilus
Other;*;Other
p__Firmicutes;*;g__Lactococcus
p__Firmicutes;*;g__Oscillospira
p__Firmicutes;*;g__
p__Firmicutes;*;g__
p__Firmicutes;*;g__
p__Firmicutes;*;g__[Eubacterium]
p__Actinobacteria;*;g__Collinsella
p__Firmicutes;*;g__
p__Firmicutes;*;g__Roseburia
p__Proteobacteria;*;g__Citrobacter
p__Firmicutes;*;g__Dorea
p__Firmicutes;*;Other
p__Proteobacteria;*;g__Bilophila
p__Firmicutes;*;g__Coprococcus
p__Firmicutes;*;g__Clostridium
p__Synergistetes;*;g__Pyramidobacter
p__Firmicutes;*;g__Holdemania
p__Firmicutes;*;g__
p__Firmicutes;*;g__Granulicatella
p__Bacteroidetes;*;g__Prevotella
p__Cyanobacteria;*;g__
p__TM7;*;g__
p__Firmicutes;*;Other
p__Actinobacteria;*;g__Eggerthella
p__Actinobacteria;*;g__Rothia
p__Firmicutes;*;g__Lactobacillus
p__TM7;*;g__
p__Actinobacteria;*;g__Actinomyces
p__Firmicutes;*;g__Coprobacillus
p__Firmicutes;*;g__Acidaminococcus
p__Firmicutes;*;g__
p__Firmicutes;*;g__Turicibacter
Accumulated abundance rank
1
50
Rank Stability Index (in %)
Rank Variability
Differences Variability
RV (50 most abundant)
DV (50 most abundant)
61.join.fna
62.join.fna
63.join.fna
64.join.fna
65.join.fna
Time samples

H5

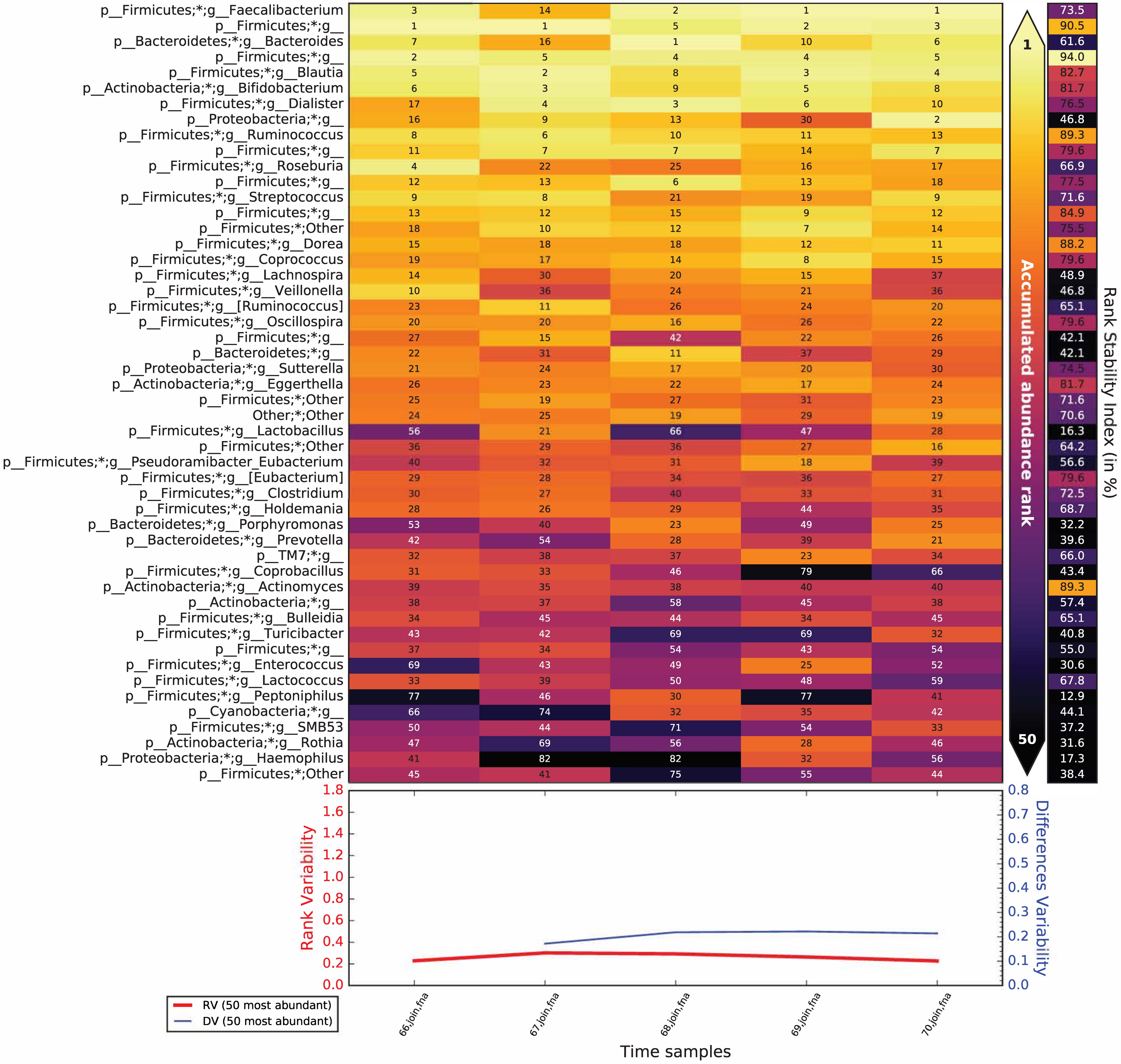

Accumulated abundance rank
1
50
Rank Stability Index (in %)
Rank Variability
Differences Variability
RV (50 most abundant)
DV (50 most abundant)
Time samples
66.join.fna
67.join.fna
68.join.fna
69.join.fna
70.join.fna
p__Firmicutes;*;g__Faecalibacterium
p__Bacteroidetes;*;g__Bacteroides
p__Firmicutes;*;g__Blautia
p__Actinobacteria;*;g__Bifidobacterium
p__Firmicutes;*;g__Dialister
p__Proteobacteria;*;g__
p__Firmicutes;*;g__Ruminococcus
p__Firmicutes;*;g__Roseburia
p__Firmicutes;*;g__Streptococcus
p__Firmicutes;*;Other
p__Firmicutes;*;g__Dorea
p__Firmicutes;*;g__Coprococcus
p__Firmicutes;*;g__Lachnospira
p__Firmicutes;*;g__Veillonella
p__Firmicutes;*;g__[Ruminococcus]
p__Firmicutes;*;g__Oscillospira
p__Bacteroidetes;*;g__
p__Proteobacteria;*;g__Sutterella
p__Actinobacteria;*;g__Eggerthella
Other;*;Other
p__Firmicutes;*;g__Lactobacillus
p__Firmicutes;*;g__Pseudoramibacter_Eubacterium
p__Firmicutes;*;g__[Eubacterium]
p__Firmicutes;*;g__Clostridium
p__Firmicutes;*;g__Holdemania
p__Bacteroidetes;*;g__Porphyromonas
p__Bacteroidetes;*;g__Prevotella
p__TM7;*;g__
p__Firmicutes;*;g__Coprobacillus
p__Actinobacteria;*;g__Actinomyces
p__Actinobacteria;*;g__
p__Firmicutes;*;g__Bulleidia
p__Firmicutes;*;g__Turicibacter
p__Firmicutes;*;g__Enterococcus
p__Firmicutes;*;g__Lactococcus
p__Firmicutes;*;g__Peptoniphilus
p__Cyanobacteria;*;g__
p__Firmicutes;*;g__SMB53
p__Actinobacteria;*;g__Rothia
p__Proteobacteria;*;g__Haemophilus

H6

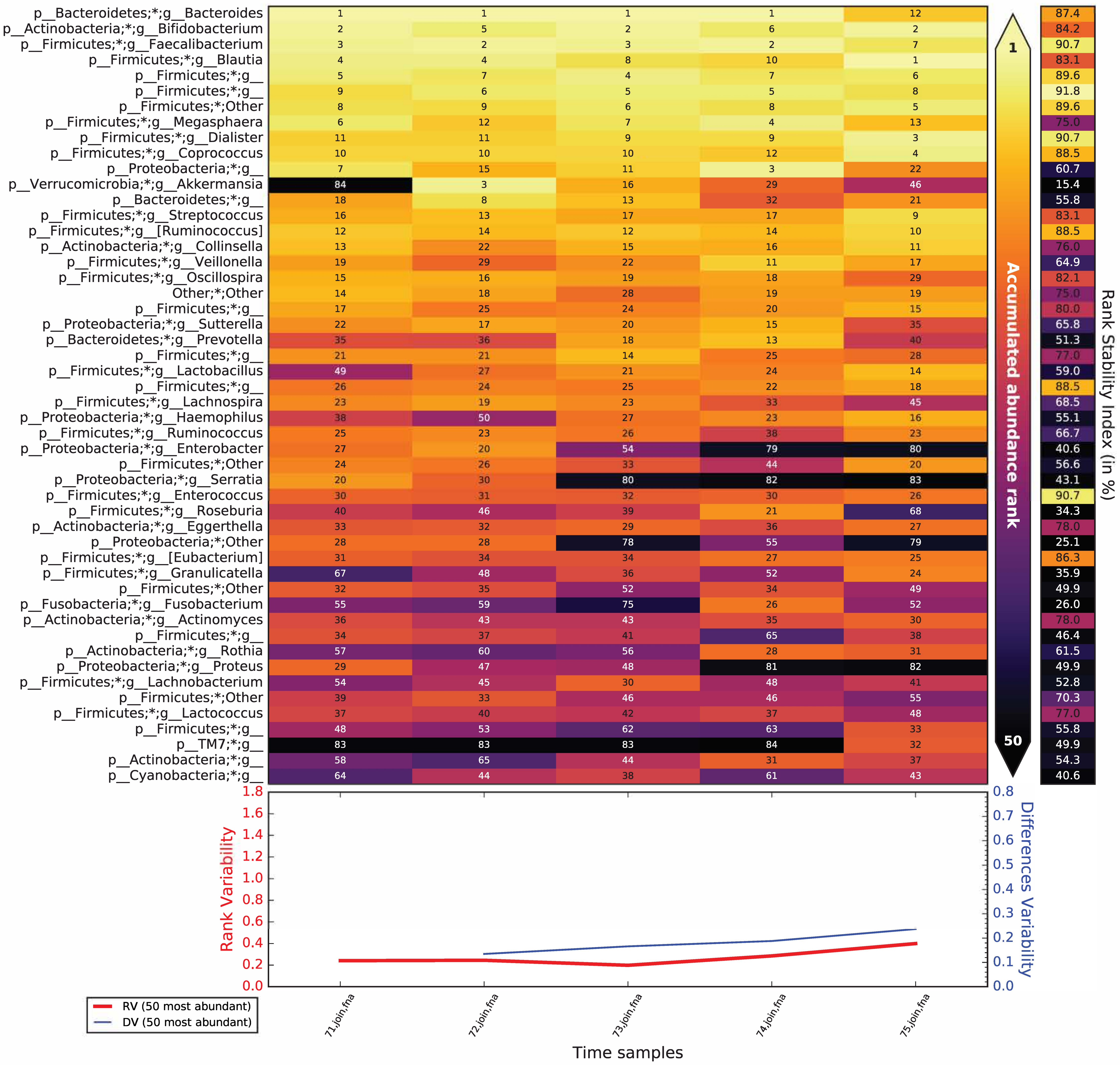
p__Bacteroidetes;*;g__Bacteroides
p__Actinobacteria;*;g__Bifidobacterium
p__Firmicutes;*;g__Faecalibacterium
p__Firmicutes;*;g__Blautia
p__Firmicutes;*;g__
p__Firmicutes;*;g__
p__Firmicutes;*;Other
p__Firmicutes;*;g__Megasphaera
p__Firmicutes;*;g__Dialister
p__Firmicutes;*;g__Coprococcus
p__Proteobacteria;*;g__
p__Verrucomicrobia;*;g__Akkermansia
p__Bacteroidetes;*;g__
p__Firmicutes;*;g__Streptococcus
p__Firmicutes;*;g__[Ruminococcus]
p__Actinobacteria;*;g__Collinsella
p__Firmicutes;*;g__Veillonella
p__Firmicutes;*;g__Oscillospira
Other;*;Other
p__Firmicutes;*;g__
p__Proteobacteria;*;g__Sutterella
p__Bacteroidetes;*;g__Prevotella
p__Firmicutes;*;g__
p__Firmicutes;*;g__Lactobacillus
p__Firmicutes;*;g__
p__Firmicutes;*;g__Lachnospira
p__Proteobacteria;*;g__Haemophilus
p__Firmicutes;*;g__Ruminococcus
p__Proteobacteria;*;g__Enterobacter
p__Firmicutes;*;Other
p__Proteobacteria;*;g__Serratia
p__Firmicutes;*;g__Enterococcus
p__Firmicutes;*;g__Roseburia
p__Actinobacteria;*;g__Eggerthella
p__Proteobacteria;*;Other
p__Firmicutes;*;g__[Eubacterium]
p__Firmicutes;*;g__Granulicatella
p__Firmicutes;*;Other
p__Fusobacteria;*;g__Fusobacterium
p__Actinobacteria;*;g__Actinomyces
p__Firmicutes;*;g__
p__Actinobacteria;*;g__Rothia
p__Proteobacteria;*;g__Proteus
p__Firmicutes;*;g__Lachnobacterium
p__Firmicutes;*;Other
p__Firmicutes;*;g__Lactococcus
p__Firmicutes;*;g__
p__TM7;*;g__
p__Actinobacteria;*;g__
p__Cyanobacteria;*;g__
Accumulated abundance rank
1
50
Rank Stability Index (in %)
Rank Variability
Differences Variability
RV (50 most abundant)
DV (50 most abundant)
71.join.fna
72.join.fna
73.join.fna
74.join.fna
75.join.fna
Time samples

C1

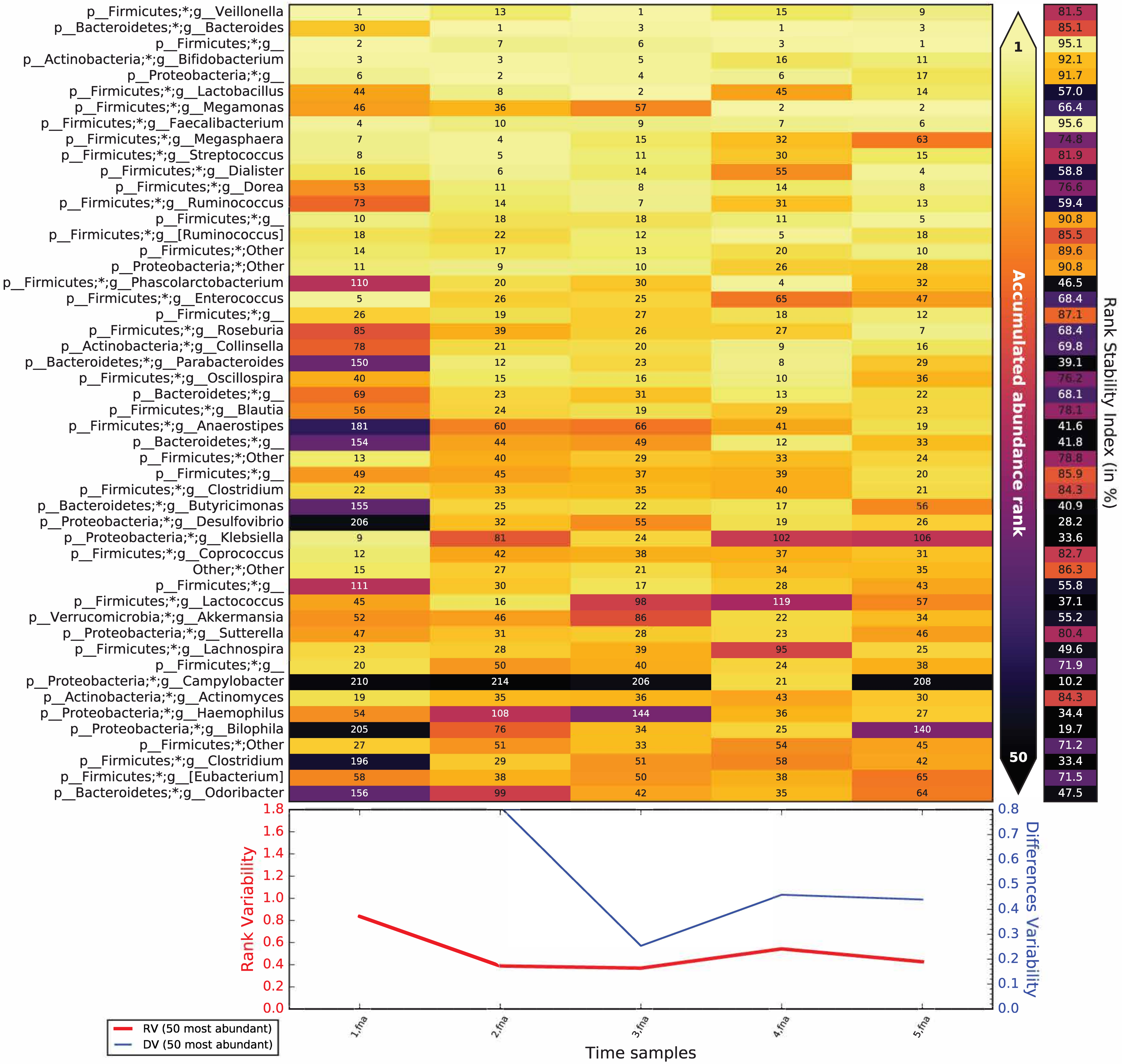
p__Firmicutes;*;g__Veillonella 1 13 1 15 9 81.5
p__Bacteroidetes;*;g__Bacteroides 30 1 3 1 3 85.1
p__Firmicutes;*;g__ 2 7 6 3 1 95.1
p__Actinobacteria;*;g__Bifidobacterium 3 3 5 16 11 92.1
p__Proteobacteria;*;g__ 6 2 4 6 17 91.7
p__Firmicutes;*;g__Lactobacillus 44 8 2 45 14 57.0
p__Firmicutes;*;g__Megamonas 46 36 57 2 2 66.4
p__Firmicutes;*;g__Faecalibacterium 4 10 9 7 6 95.6
p__Firmicutes;*;g__Megasphaera 7 4 15 32 63 74.8
p__Firmicutes;*;g__Streptococcus 8 5 11 30 15 81.9
p__Firmicutes;*;g__Dialister 16 6 14 55 4 58.8
p__Firmicutes;*;g__Dorea 53 11 8 14 8 76.6
p__Firmicutes;*;g__Ruminococcus 73 14 7 31 13 59.4
p__Firmicutes;*;g__ 10 18 18 11 5 90.8
p__Firmicutes;*;g__[Ruminococcus] 18 22 12 5 18 85.5
p__Firmicutes;*;Other 14 17 13 20 10 89.6
p__Proteobacteria;*;Other 11 9 10 26 28 90.8
p__Firmicutes;*;g__Phascolarctobacterium 110 20 30 4 32 46.5
p__Firmicutes;*;g__Enterococcus 5 26 25 65 47 68.4
p__Firmicutes;*;g__ 26 19 27 18 12 87.1
p__Firmicutes;*;g__Roseburia 85 39 26 27 7 68.4
p__Actinobacteria;*;g__Collinsella 78 21 20 9 16 69.8
p__Bacteroidetes;*;g__Parabacteroides 150 12 23 8 29 39.1
p__Firmicutes;*;g__Oscillospira 40 15 16 10 36 76.2
p__Bacteroidetes;*;g__ 69 23 31 13 22 68.1
p__Firmicutes;*;g__Blautia 56 24 19 29 23 78.1
p__Firmicutes;*;g__Anaerostipes 181 60 66 41 19 41.6
p__Bacteroidetes;*;g__ 154 44 49 12 33 41.8
p__Firmicutes;*;Other 13 40 29 33 24 78.8
p__Firmicutes;*;g__ 49 45 37 39 20 85.9
p__Firmicutes;*;g__Clostridium 22 33 35 40 21 84.3
p__Bacteroidetes;*;g__Butyricimonas 155 25 22 17 56 40.9
p__Proteobacteria;*;g__Desulfovibrio 206 32 55 19 26 28.2
p__Proteobacteria;*;g__Klebsiella 9 81 24 102 106 33.6
p__Firmicutes;*;g__Coprococcus 12 42 38 37 31 82.7
Other;*;Other 15 27 21 34 35 86.3
p__Firmicutes;*;g__ 111 30 17 28 43 55.8
p__Firmicutes;*;g__Lactococcus 45 16 98 119 57 37.1
p__Verrucomicrobia;*;g__Akkermansia 52 46 86 22 34 55.2
p__Proteobacteria;*;g__Sutterella 47 31 28 23 46 80.4
p__Firmicutes;*;g__Lachnospira 23 28 39 95 25 49.6
p__Firmicutes;*;g__ 20 50 40 24 38 71.9
p__Proteobacteria;*;g__Campylobacter 210 214 206 21 208 10.2
p__Actinobacteria;*;g__Actinomyces 19 35 36 43 30 84.3
p__Proteobacteria;*;g__Haemophilus 54 108 144 36 27 34.4
p__Proteobacteria;*;g__Bilophila 205 76 34 25 140 19.7
p__Firmicutes;*;Other 27 51 33 54 45 71.2
p__Firmicutes;*;g__Clostridium 196 29 51 58 42 33.4
p__Firmicutes;*;g__[Eubacterium] 58 38 50 38 65 71.5
p__Bacteroidetes;*;g__Odoribacter 156 99 42 35 64 47.5
1
Accumulated abundance rank
50
Rank Stability Index (in %)
Rank Variability
1.8
1.6
1.4
1.2
1.0
0.8
0.6
0.4
0.2
0.0
Differences Variability
0.8
0.7
0.6
0.5
0.4
0.3
0.2
0.1
0.0
1.fna
2.fna
3.fna
4.fna
5.fna
Time samples
RV (50 most abundant)
DV (50 most abundant)

C2

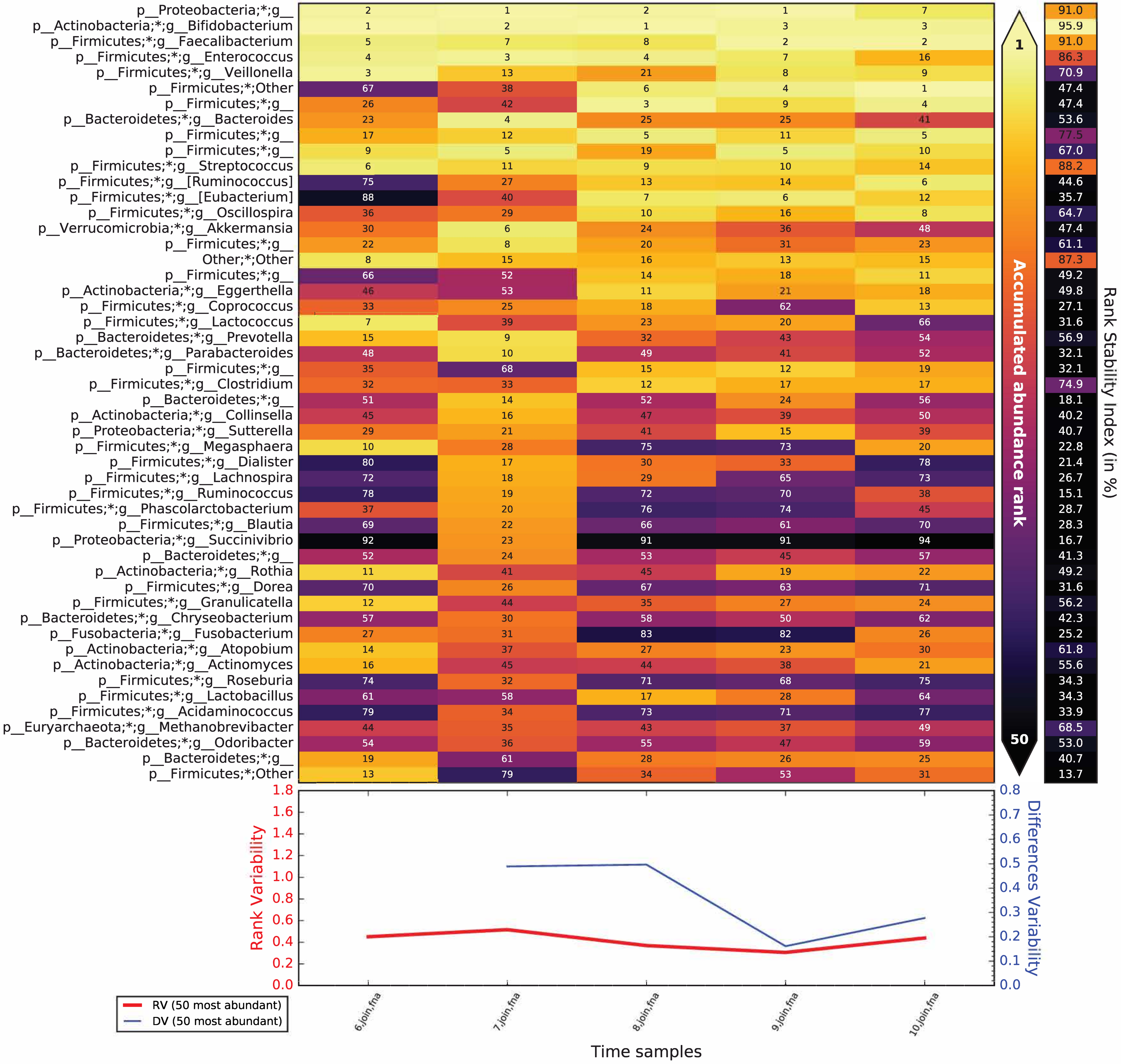
p__Proteobacteria;*;g__
p__Actinobacteria;*;g__Bifidobacterium
p__Firmicutes;*;g__Faecalibacterium
p__Firmicutes;*;g__Enterococcus
p__Firmicutes;*;g__Veillonella
p__Firmicutes;*;Other
p__Firmicutes;*;g__
p__Bacteroidetes;*;g__Bacteroides
p__Firmicutes;*;g__
p__Firmicutes;*;g__
p__Firmicutes;*;g__Streptococcus
p__Firmicutes;*;g__[Ruminococcus]
p__Firmicutes;*;g__[Eubacterium]
p__Firmicutes;*;g__Oscillospira
p__Verrucomicrobia;*;g__Akkermansia
p__Firmicutes;*;g__
Other;*;Other
p__Firmicutes;*;g__
p__Actinobacteria;*;g__Eggerthella
p__Firmicutes;*;g__Coprococcus
p__Firmicutes;*;g__Lactococcus
p__Bacteroidetes;*;g__Prevotella
p__Bacteroidetes;*;g__Parabacteroides
p__Firmicutes;*;g__
p__Firmicutes;*;g__Clostridium
p__Bacteroidetes;*;g__
p__Actinobacteria;*;g__Collinsella
p__Proteobacteria;*;g__Sutterella
p__Firmicutes;*;g__Megasphaera
p__Firmicutes;*;g__Dialister
p__Firmicutes;*;g__Lachnospira
p__Firmicutes;*;g__Ruminococcus
p__Firmicutes;*;g__Phascolarctobacterium
p__Firmicutes;*;g__Blautia
p__Proteobacteria;*;g__Succinivibrio
p__Bacteroidetes;*;g__
p__Actinobacteria;*;g__Rothia
p__Firmicutes;*;g__Dorea
p__Firmicutes;*;g__Granulicatella
p__Bacteroidetes;*;g__Chryseobacterium
p__Fusobacteria;*;g__Fusobacterium
p__Actinobacteria;*;g__Atopobium
p__Actinobacteria;*;g__Actinomyces
p__Firmicutes;*;g__Roseburia
p__Firmicutes;*;g__Lactobacillus
p__Firmicutes;*;g__Acidaminococcus
p__Euryarchaeota;*;g__Methanobrevibacter
p__Bacteroidetes;*;g__Odoribacter
p__Bacteroidetes;*;g__
p__Firmicutes;*;Other
Accumulated abundance rank
1
50
Rank Stability Index (in %)
91.0
95.9
91.0
86.3
70.9
47.4
47.4
53.6
77.5
67.0
88.2
44.6
35.7
64.7
47.4
61.1
87.3
49.2
49.8
27.1
31.6
56.9
32.1
32.1
74.9
18.1
40.2
40.7
22.8
21.4
26.7
15.1
28.7
28.3
16.7
41.3
49.2
31.6
56.2
42.3
25.2
61.8
55.6
34.3
34.3
33.9
68.5
53.0
40.7
13.7
Rank Variability
Differences Variability
RV (50 most abundant)
DV (50 most abundant)
6.join.fna
7.join.fna
8.join.fna
9.join.fna
10.join.fna
Time samples

C3

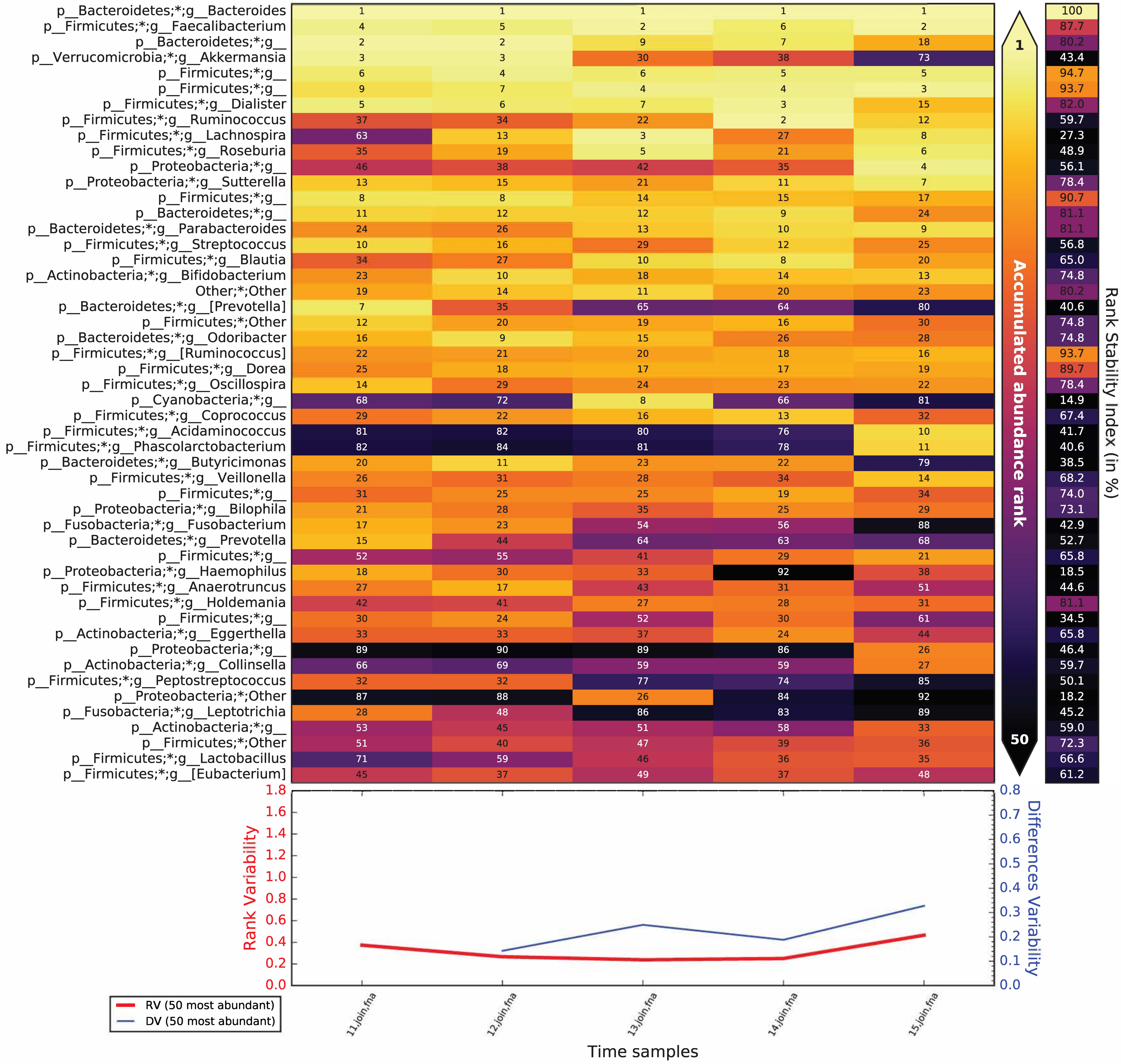
p__Bacteroidetes;*;g__Bacteroides 1 1 1 1 1 100
p__Firmicutes;*;g__Faecalibacterium 4 5 2 6 2 87.7
p__Bacteroidetes;*;g__ 2 2 9 7 18 80.2
p__Verrucomicrobia;*;g__Akkermansia 3 3 30 38 73 43.4
p__Firmicutes;*;g__ 6 4 6 5 5 94.7
p__Firmicutes;*;g__ 9 7 4 4 3 93.7
p__Firmicutes;*;g__Dialister 5 6 7 3 15 82.0
p__Firmicutes;*;g__Ruminococcus 37 34 22 2 12 59.7
p__Firmicutes;*;g__Lachnospira 63 13 3 27 8 27.3
p__Firmicutes;*;g__Roseburia 35 19 5 21 6 48.9
p__Proteobacteria;*;g__ 46 38 42 35 4 56.1
p__Proteobacteria;*;g__Sutterella 13 15 21 11 7 78.4
p__Firmicutes;*;g__ 8 8 14 15 17 90.7
p__Bacteroidetes;*;g__ 11 12 12 9 24 81.1
p__Bacteroidetes;*;g__Parabacteroides 24 26 13 10 9 81.1
p__Firmicutes;*;g__Streptococcus 10 16 29 12 25 56.8
p__Firmicutes;*;g__Blautia 34 27 10 8 20 65.0
p__Actinobacteria;*;g__Bifidobacterium 23 10 18 14 13 74.8
Other;*;Other 19 14 11 20 23 80.2
p__Bacteroidetes;*;g__[Prevotella] 7 35 65 64 80 40.6
p__Firmicutes;*;Other 12 20 19 16 30 74.8
p__Bacteroidetes;*;g__Odoribacter 16 9 15 26 28 74.8
p__Firmicutes;*;g__[Ruminococcus] 22 21 20 18 16 93.7
p__Firmicutes;*;g__Dorea 25 18 17 17 19 89.7
p__Firmicutes;*;g__Oscillospira 14 29 24 23 22 78.4
p__Cyanobacteria;*;g__ 68 72 8 66 81 14.9
p__Firmicutes;*;g__Coprococcus 29 22 16 13 32 67.4
p__Firmicutes;*;g__Acidaminococcus 81 82 80 76 10 41.7
p__Firmicutes;*;g__Phascolarctobacterium 82 84 81 78 11 40.6
p__Bacteroidetes;*;g__Butyricimonas 20 11 23 22 79 38.5
p__Firmicutes;*;g__Veillonella 26 31 28 34 14 68.2
p__Firmicutes;*;g__ 31 25 25 19 34 74.0
p__Proteobacteria;*;g__Bilophila 21 28 35 25 29 73.1
p__Fusobacteria;*;g__Fusobacterium 17 23 54 56 88 42.9
p__Bacteroidetes;*;g__Prevotella 15 44 64 63 68 52.7
p__Firmicutes;*;g__ 52 55 41 29 21 65.8
p__Proteobacteria;*;g__Haemophilus 18 30 33 92 38 18.5
p__Firmicutes;*;g__Anaerotruncus 27 17 43 31 51 44.6
p__Firmicutes;*;g__Holdemania 42 41 27 28 31 81.1
p__Firmicutes;*;g__ 30 24 52 30 61 34.5
p__Actinobacteria;*;g__Eggerthella 33 33 37 24 44 65.8
p__Proteobacteria;*;g__ 89 90 89 86 26 46.4
p__Actinobacteria;*;g__Collinsella 66 69 59 59 27 59.7
p__Firmicutes;*;g__Peptostreptococcus 32 32 77 74 85 50.1
p__Proteobacteria;*;Other 87 88 26 84 92 18.2
p__Fusobacteria;*;g__Leptotrichia 28 48 86 83 89 45.2
p__Actinobacteria;*;g__ 53 45 51 58 33 59.0
p__Firmicutes;*;Other 51 40 47 39 36 72.3
p__Firmicutes;*;g__Lactobacillus 71 59 46 36 35 66.6
p__Firmicutes;*;g__[Eubacterium] 45 37 49 37 48 61.2
1
Accumulated abundance rank
50
Rank Stability Index (in %)
Rank Variability
1.8
1.6
1.4
1.2
1.0
0.8
0.6
0.4
0.2
0.0
Differences Variability
0.8
0.7
0.6
0.5
0.4
0.3
0.2
0.1
0.0
RV (50 most abundant)
DV (50 most abundant)
11.join.fna
12.join.fna
13.join.fna
14.join.fna
15.join.fna
Time samples

C4

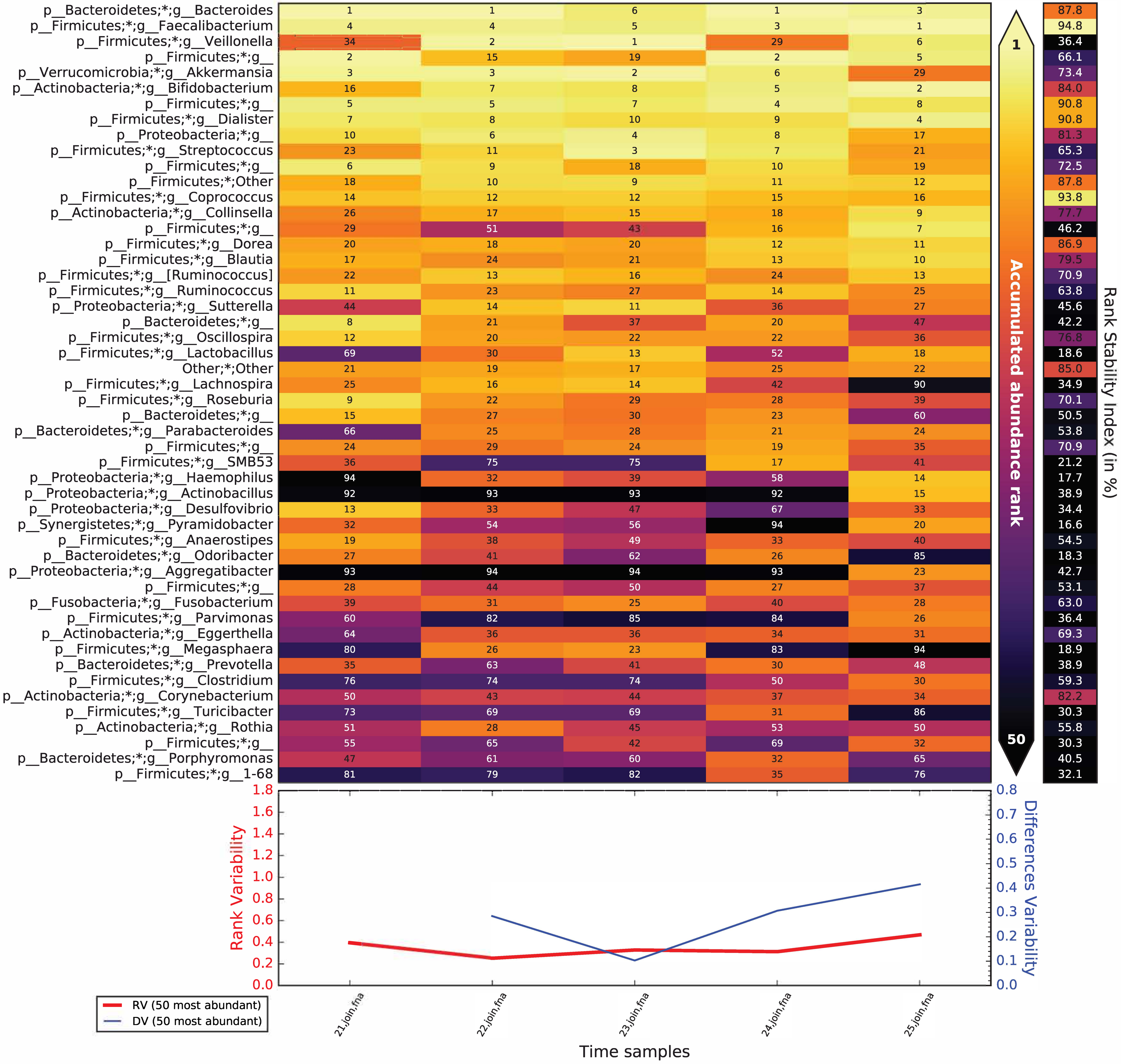
Accumulated abundance rank
Rank Stability Index (in %)
Rank Variability
Differences Variability
Time samples
RV (50 most abundant)
DV (50 most abundant)
21.join.fna
22.join.fna
23.join.fna
24.join.fna
25.join.fna

C5

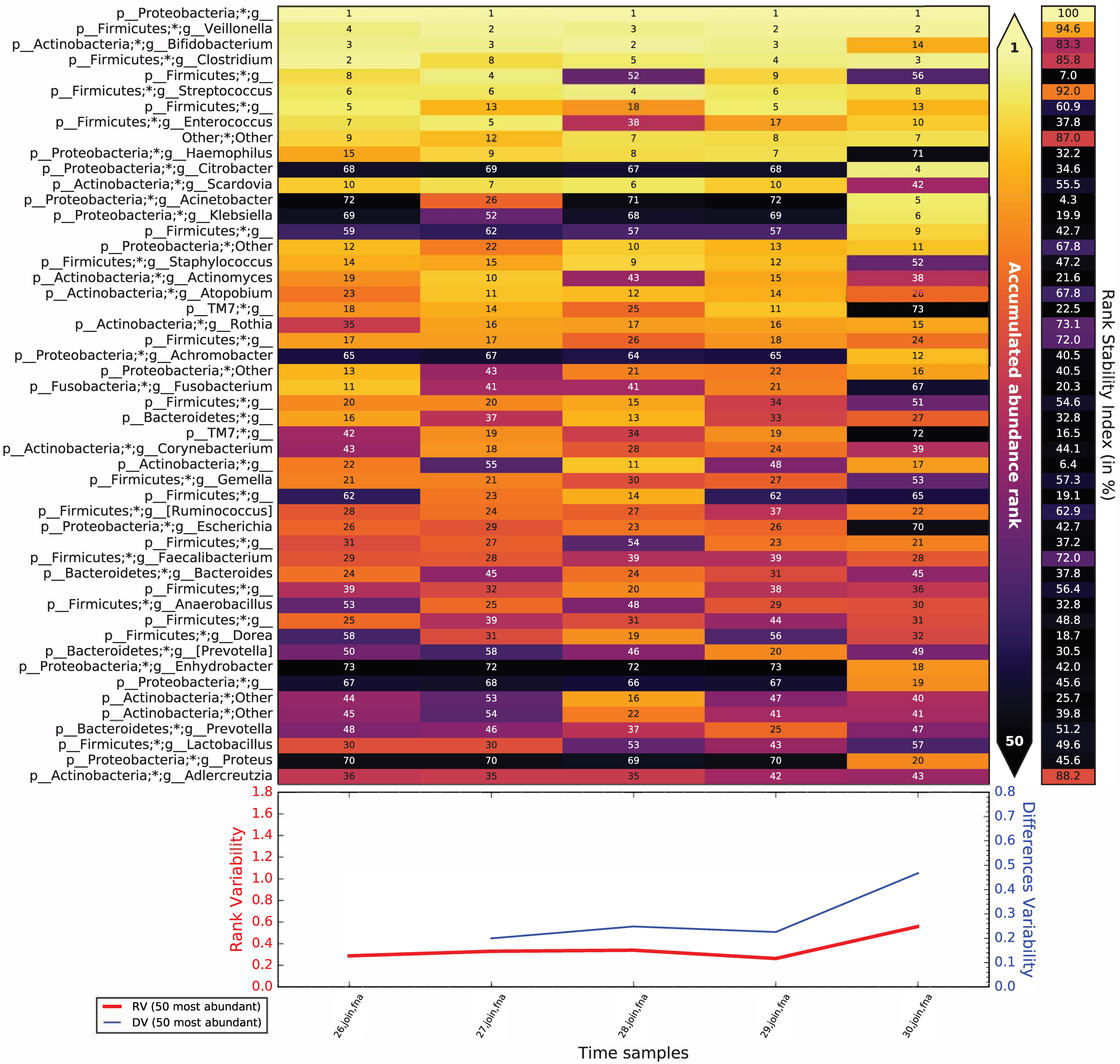

p__Proteobacteria;*;g__
p__Firmicutes;*;g__Veillonella
p__Actinobacteria;*;g__Bifidobacterium
p__Firmicutes;*;g__Clostridium
p__Firmicutes;*;g__
p__Firmicutes;*;g__Streptococcus
p__Firmicutes;*;g__
p__Firmicutes;*;g__Enterococcus
Other;*;Other
p__Proteobacteria;*;g__Haemophilus
p__Proteobacteria;*;g__Citrobacter
p__Actinobacteria;*;g__Scardovia
p__Proteobacteria;*;g__Acinetobacter
p__Proteobacteria;*;g__Klebsiella
p__Firmicutes;*;g__
p__Proteobacteria;*;Other
p__Firmicutes;*;g__Staphylococcus
p__Actinobacteria;*;g__Actinomyces
p__Actinobacteria;*;g__Atopobium
p__TM7;*;g__
p__Actinobacteria;*;g__Rothia
p__Firmicutes;*;g__
p__Proteobacteria;*;g__Achromobacter
p__Proteobacteria;*;Other
p__Fusobacteria;*;g__Fusobacterium
p__Firmicutes;*;g__
p__Bacteroidetes;*;g__
p__TM7;*;g__
p__Actinobacteria;*;g__Corynebacterium
p__Actinobacteria;*;g__
p__Firmicutes;*;g__Gemella
p__Firmicutes;*;g__
p__Firmicutes;*;g__[Ruminococcus]
p__Proteobacteria;*;g__Escherichia
p__Firmicutes;*;g__
p__Firmicutes;*;g__Faecalibacterium
p__Bacteroidetes;*;g__Bacteroides
p__Firmicutes;*;g__
p__Firmicutes;*;g__Anaerobacillus
p__Firmicutes;*;g__
p__Firmicutes;*;g__Dorea
p__Bacteroidetes;*;g__[Prevotella]
p__Proteobacteria;*;g__Enhydrobacter
p__Proteobacteria;*;g__
p__Actinobacteria;*;Other
p__Actinobacteria;*;Other
p__Bacteroidetes;*;g__Prevotella
p__Firmicutes;*;g__Lactobacillus
p__Proteobacteria;*;g__Proteus
p__Actinobacteria;*;g__Adlercreutzia
Accumulated abundance rank
1
50
Rank Stability Index (in %)
100
94.6
83.3
85.8
7.0
92.0
60.9
37.8
87.0
32.2
34.6
55.5
4.3
19.9
42.7
67.8
47.2
21.6
67.8
22.5
73.1
72.0
40.5
40.5
20.3
54.6
32.8
16.5
44.1
6.4
57.3
19.1
62.9
42.7
37.2
72.0
37.8
56.4
32.8
48.8
18.7
30.5
42.0
45.6
25.7
39.8
51.2
49.6
45.6
88.2
Rank Variability
Differences Variability
RV (50 most abundant)
DV (50 most abundant)
26.join.fna
27.join.fna
28.join.fna
29.join.fna
30.join.fna
Time samples

C6

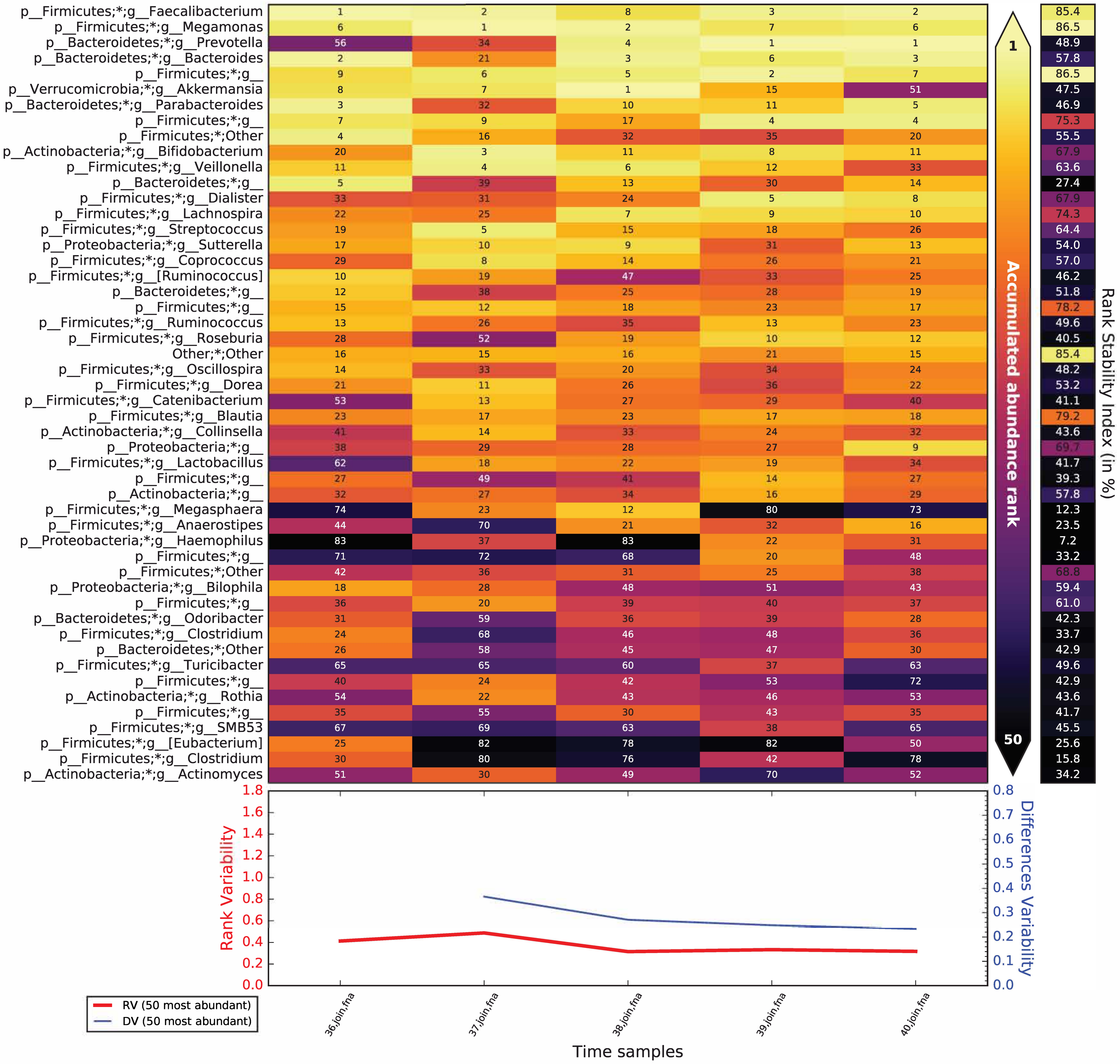
p__Firmicutes;*;g__Faecalibacterium 1 2 8 3 2 85.4
p__Firmicutes;*;g__Megamonas 6 1 2 7 6 86.5
p__Bacteroidetes;*;g__Prevotella 56 34 4 1 1 48.9
p__Bacteroidetes;*;g__Bacteroides 2 21 3 6 3 57.8
p__Firmicutes;*;g__ 9 6 5 2 7 86.5
p__Verrucomicrobia;*;g__Akkermansia 8 7 1 15 51 47.5
p__Bacteroidetes;*;g__Parabacteroides 3 32 10 11 5 46.9
p__Firmicutes;*;g__ 7 9 17 4 4 75.3
p__Firmicutes;*;Other 4 16 32 35 20 55.5
p__Actinobacteria;*;g__Bifidobacterium 20 3 11 8 11 67.9
p__Firmicutes;*;g__Veillonella 11 4 6 12 33 63.6
p__Bacteroidetes;*;g__ 5 39 13 30 14 27.4
p__Firmicutes;*;g__Dialister 33 31 24 5 8 67.9
p__Firmicutes;*;g__Lachnospira 22 25 7 9 10 74.3
p__Firmicutes;*;g__Streptococcus 19 5 15 18 26 64.4
p__Proteobacteria;*;g__Sutterella 17 10 9 31 13 54.0
p__Firmicutes;*;g__Coprococcus 29 8 14 26 21 57.0
p__Firmicutes;*;g__[Ruminococcus] 10 19 47 33 25 46.2
p__Bacteroidetes;*;g__ 12 38 25 28 19 51.8
p__Firmicutes;*;g__ 15 12 18 23 17 78.2
p__Firmicutes;*;g__Ruminococcus 13 26 35 13 23 49.6
p__Firmicutes;*;g__Roseburia 28 52 19 10 12 40.5
Other;*;Other 16 15 16 21 15 85.4
p__Firmicutes;*;g__Oscillospira 14 33 20 34 24 48.2
p__Firmicutes;*;g__Dorea 21 11 26 36 22 53.2
p__Firmicutes;*;g__Catenibacterium 53 13 27 29 40 41.1
p__Firmicutes;*;g__Blautia 23 17 23 17 18 79.2
p__Actinobacteria;*;g__Collinsella 41 14 33 24 32 43.6
p__Proteobacteria;*;g__ 38 29 28 27 9 69.7
p__Firmicutes;*;g__Lactobacillus 62 18 22 19 34 41.7
p__Firmicutes;*;g__ 27 49 41 14 27 39.3
p__Actinobacteria;*;g__ 32 27 34 16 29 57.8
p__Firmicutes;*;g__Megasphaera 74 23 12 80 73 12.3
p__Firmicutes;*;g__Anaerostipes 44 70 21 32 16 23.5
p__Proteobacteria;*;g__Haemophilus 83 37 83 22 31 7.2
p__Firmicutes;*;g__ 71 72 68 20 48 33.2
p__Firmicutes;*;Other 42 36 31 25 38 68.8
p__Proteobacteria;*;g__Bilophila 18 28 48 51 43 59.4
p__Firmicutes;*;g__ 36 20 39 40 37 61.0
p__Bacteroidetes;*;g__Odoribacter 31 59 36 39 28 42.3
p__Firmicutes;*;g__Clostridium 24 68 46 48 36 33.7
p__Bacteroidetes;*;Other 26 58 45 47 30 42.9
p__Firmicutes;*;g__Turicibacter 65 65 60 37 63 49.6
p__Firmicutes;*;g__ 40 24 42 53 72 42.9
p__Actinobacteria;*;g__Rothia 54 22 43 46 53 43.6
p__Firmicutes;*;g__ 35 55 30 43 35 41.7
p__Firmicutes;*;g__SMB53 67 69 63 38 65 45.5
p__Firmicutes;*;g__[Eubacterium] 25 82 78 82 50 25.6
p__Firmicutes;*;g__Clostridium 30 80 76 42 78 15.8
p__Actinobacteria;*;g__Actinomyces 51 30 49 70 52 34.2
1
Accumulated abundance rank
50
Rank Stability Index (in %)
Rank Variability
1.8 1.6 1.4 1.2 1.0 0.8 0.6 0.4 0.2 0.0
Differences Variability
0.8 0.7 0.6 0.5 0.4 0.3 0.2 0.1 0.0
RV (50 most abundant)
DV (50 most abundant)
36.join.fna
37.join.fna
38.join.fna
39.join.fna
40.join.fna
Time samples

C7

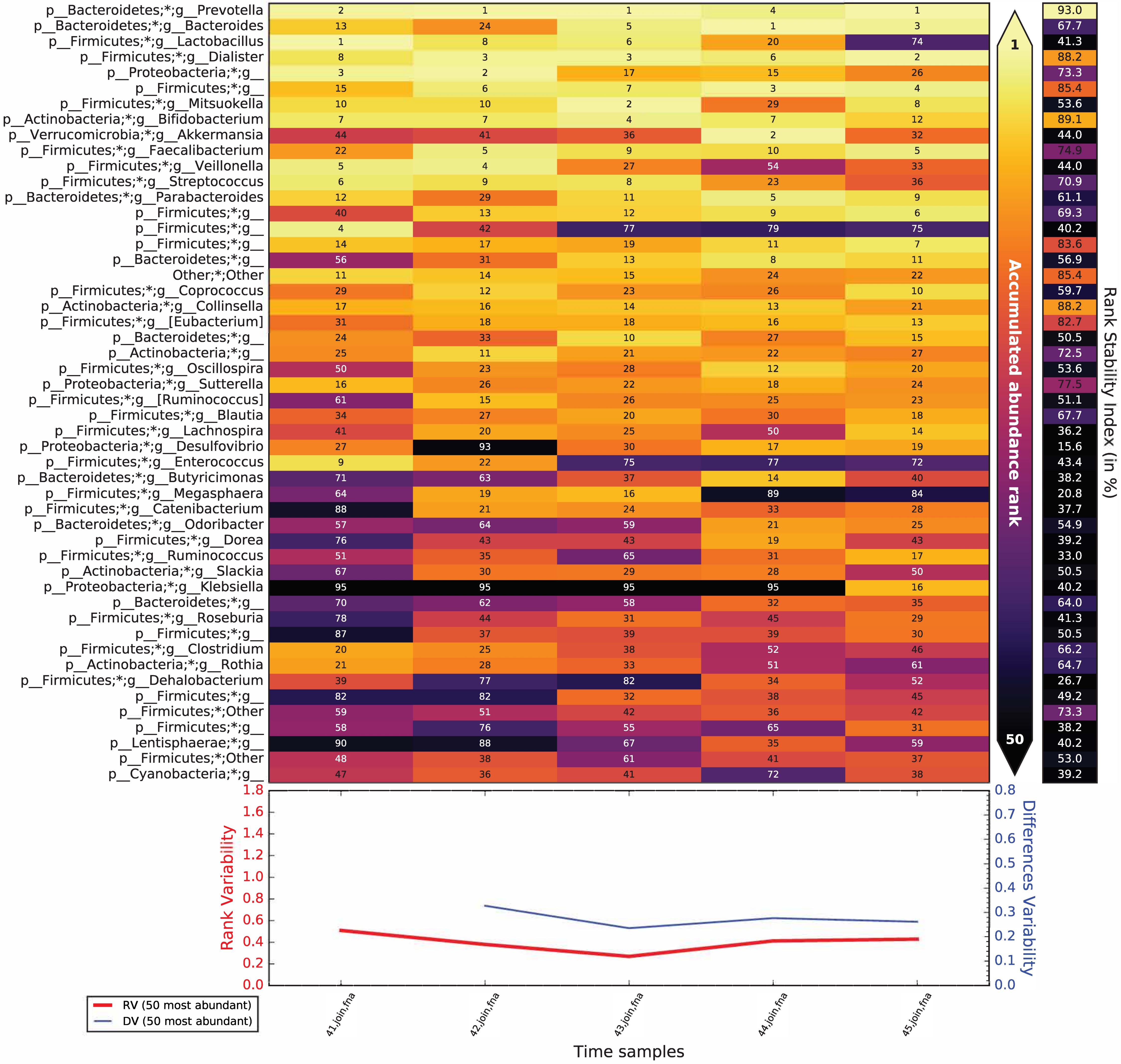
p__Bacteroidetes;*;g__Prevotella 2 1 1 4 1 93.0
p__Bacteroidetes;*;g__Bacteroides 13 24 5 1 3 67.7
p__Firmicutes;*;g__Lactobacillus 1 8 6 20 74 41.3
p__Firmicutes;*;g__Dialister 8 3 3 6 2 88.2
p__Proteobacteria;*;g__ 3 2 17 15 26 73.3
p__Firmicutes;*;g__ 15 6 7 3 4 85.4
p__Firmicutes;*;g__Mitsuokella 10 10 2 29 8 53.6
p__Actinobacteria;*;g__Bifidobacterium 7 7 4 7 12 89.1
p__Verrucomicrobia;*;g__Akkermansia 44 41 36 2 32 44.0
p__Firmicutes;*;g__Faecalibacterium 22 5 9 10 5 74.9
p__Firmicutes;*;g__Veillonella 5 4 27 54 33 44.0
p__Firmicutes;*;g__Streptococcus 6 9 8 23 36 70.9
p__Bacteroidetes;*;g__Parabacteroides 12 29 11 5 9 61.1
p__Firmicutes;*;g__ 40 13 12 9 6 69.3
p__Firmicutes;*;g__ 4 42 77 79 75 40.2
p__Firmicutes;*;g__ 14 17 19 11 7 83.6
p__Bacteroidetes;*;g__ 56 31 13 8 11 56.9
Other;*;Other 11 14 15 24 22 85.4
p__Firmicutes;*;g__Coprococcus 29 12 23 26 10 59.7
p__Actinobacteria;*;g__Collinsella 17 16 14 13 21 88.2
p__Firmicutes;*;g__[Eubacterium] 31 18 18 16 13 82.7
p__Bacteroidetes;*;g__ 24 33 10 27 15 50.5
p__Actinobacteria;*;g__ 25 11 21 22 27 72.5
p__Firmicutes;*;g__Oscillospira 50 23 28 12 20 53.6
p__Proteobacteria;*;g__Sutterella 16 26 22 18 24 77.5
p__Firmicutes;*;g__[Ruminococcus] 61 15 26 25 23 51.1
p__Firmicutes;*;g__Blautia 34 27 20 30 18 67.7
p__Firmicutes;*;g__Lachnospira 41 20 25 50 14 36.2
p__Proteobacteria;*;g__Desulfovibrio 27 93 30 17 19 15.6
p__Firmicutes;*;g__Enterococcus 9 22 75 77 72 43.4
p__Bacteroidetes;*;g__Butyricimonas 71 63 37 14 40 38.2
p__Firmicutes;*;g__Megasphaera 64 19 16 89 84 20.8
p__Firmicutes;*;g__Catenibacterium 88 21 24 33 28 37.7
p__Bacteroidetes;*;g__Odoribacter 57 64 59 21 25 54.9
p__Firmicutes;*;g__Dorea 76 43 43 19 43 39.2
p__Firmicutes;*;g__Ruminococcus 51 35 65 31 17 33.0
p__Actinobacteria;*;g__Slackia 67 30 29 28 50 50.5
p__Proteobacteria;*;g__Klebsiella 95 95 95 95 16 40.2
p__Bacteroidetes;*;g__ 70 62 58 32 35 64.0
p__Firmicutes;*;g__Roseburia 78 44 31 45 29 41.3
p__Firmicutes;*;g__ 87 37 39 39 30 50.5
p__Firmicutes;*;g__Clostridium 20 25 38 52 46 66.2
p__Actinobacteria;*;g__Rothia 21 28 33 51 61 64.7
p__Firmicutes;*;g__Dehalobacterium 39 77 82 34 52 26.7
p__Firmicutes;*;g__ 82 82 32 38 45 49.2
p__Firmicutes;*;Other 59 51 42 36 42 73.3
p__Firmicutes;*;g__ 58 76 55 65 31 38.2
p__Lentisphaerae;*;g__ 90 88 67 35 59 40.2
p__Firmicutes;*;Other 48 38 61 41 37 53.0
p__Cyanobacteria;*;g__ 47 36 41 72 38 39.2
1
50
Accumulated abundance rank
Rank Stability Index (in %)
Rank Variability
Differences Variability
1.8 1.6 1.4 1.2 1.0 0.8 0.6 0.4 0.2 0.0
0.8 0.7 0.6 0.5 0.4 0.3 0.2 0.1 0.0
RV (50 most abundant)
DV (50 most abundant)
41.join.fna
42.join.fna
43.join.fna
44.join.fna
45.join.fna
Time samples

C8

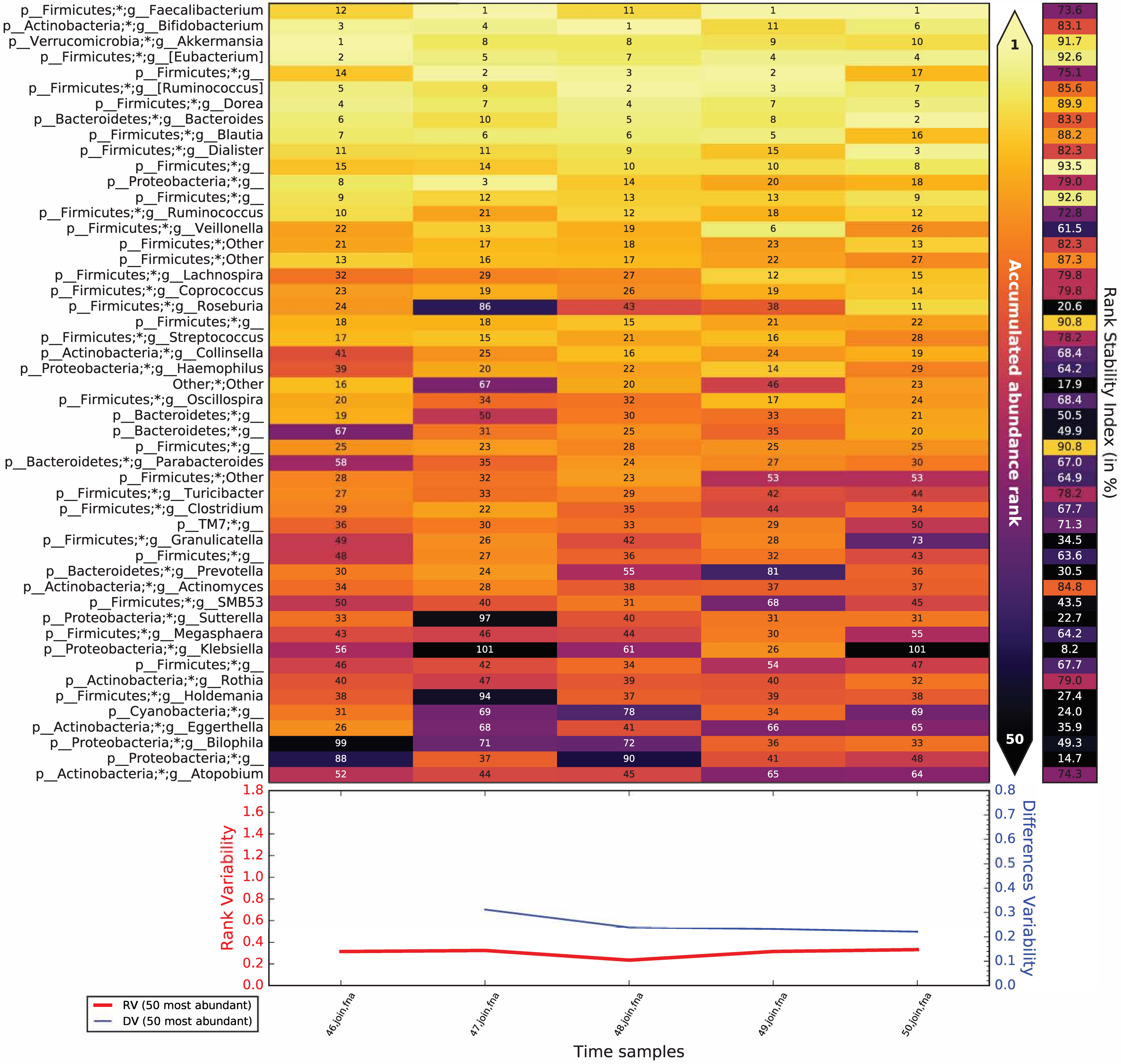
p__Firmicutes;*;g__Faecalibacterium
p__Actinobacteria;*;g__Bifidobacterium
p__Verrucomicrobia;*;g__Akkermansia
p__Firmicutes;*;g__[Eubacterium]
p__Firmicutes;*;g__
p__Firmicutes;*;g__[Ruminococcus]
p__Firmicutes;*;g__Dorea
p__Bacteroidetes;*;g__Bacteroides
p__Firmicutes;*;g__Blautia
p__Firmicutes;*;g__Dialister
p__Firmicutes;*;g__
p__Proteobacteria;*;g__
p__Firmicutes;*;g__
p__Firmicutes;*;g__Ruminococcus
p__Firmicutes;*;g__Veillonella
p__Firmicutes;*;Other
p__Firmicutes;*;Other
p__Firmicutes;*;g__Lachnospira
p__Firmicutes;*;g__Coprococcus
p__Firmicutes;*;g__Roseburia
p__Firmicutes;*;g__
p__Firmicutes;*;g__Streptococcus
p__Actinobacteria;*;g__Collinsella
p__Proteobacteria;*;g__Haemophilus
Other;*;Other
p__Firmicutes;*;g__Oscillospira
p__Bacteroidetes;*;g__
p__Bacteroidetes;*;g__
p__Firmicutes;*;g__
p__Bacteroidetes;*;g__Parabacteroides
p__Firmicutes;*;Other
p__Firmicutes;*;g__Turicibacter
p__Firmicutes;*;g__Clostridium
p__TM7;*;g__
p__Firmicutes;*;g__Granulicatella
p__Firmicutes;*;g__
p__Bacteroidetes;*;g__Prevotella
p__Actinobacteria;*;g__Actinomyces
p__Firmicutes;*;g__SMB53
p__Proteobacteria;*;g__Sutterella
p__Firmicutes;*;g__Megasphaera
p__Proteobacteria;*;g__Klebsiella
p__Firmicutes;*;g__
p__Actinobacteria;*;g__Rothia
p__Firmicutes;*;g__Holdemania
p__Cyanobacteria;*;g__
p__Actinobacteria;*;g__Eggerthella
p__Proteobacteria;*;g__Bilophila
p__Proteobacteria;*;g__
p__Actinobacteria;*;g__Atopobium
Accumulated abundance rank
1
50
Rank Stability Index (in %)
Rank Variability
Differences Variability
RV (50 most abundant)
DV (50 most abundant)
46.join.fna
47.join.fna
48.join.fna
49.join.fna
50.join.fna
Time samples

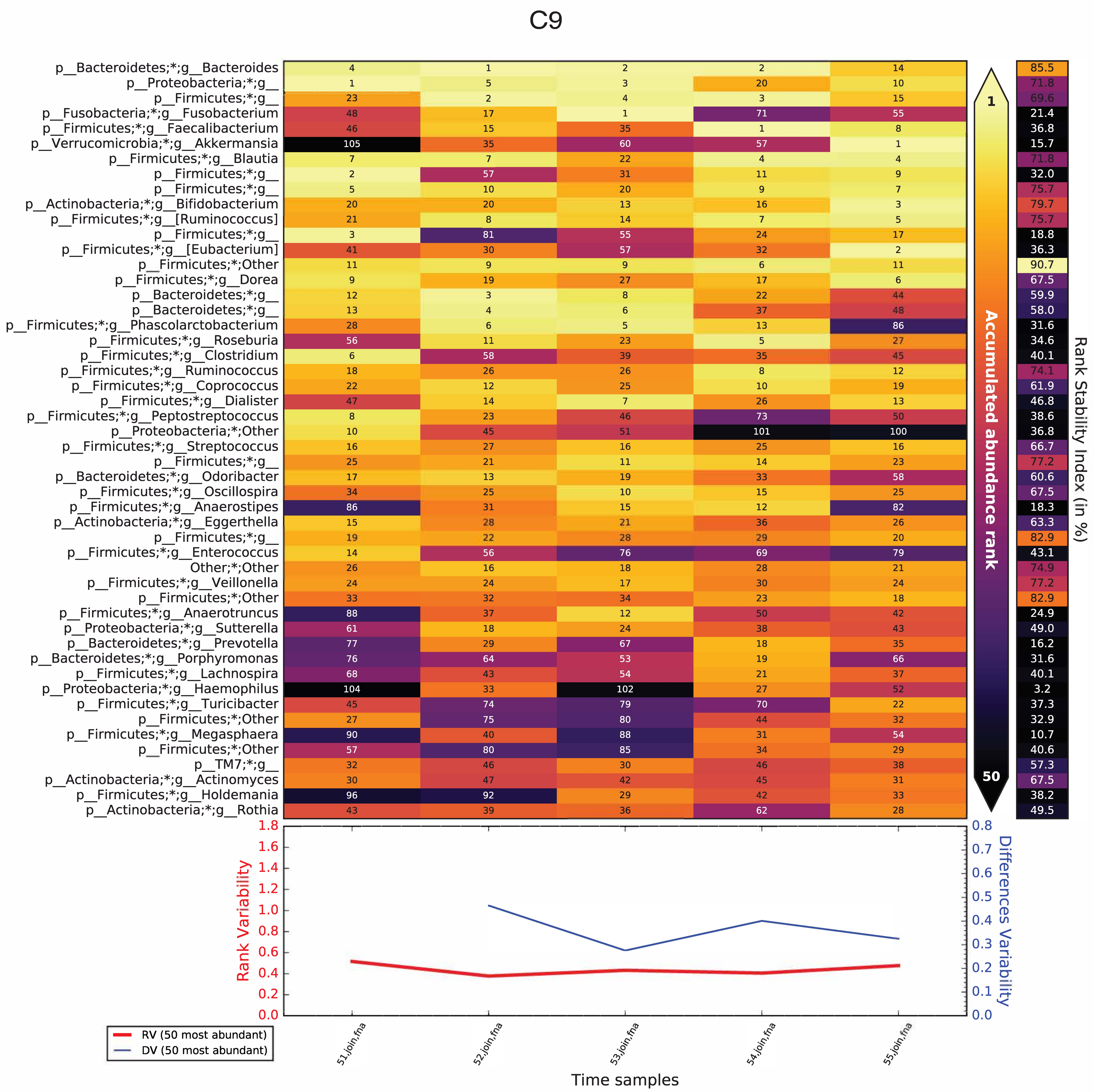

C9
p__Bacteroidetes;*;g__Bacteroides
p__Proteobacteria;*;g__
p__Firmicutes;*;g__
p__Fusobacteria;*;g__Fusobacterium
p__Firmicutes;*;g__Faecalibacterium
p__Verrucomicrobia;*;g__Akkermansia
p__Firmicutes;*;g__Blautia
p__Actinobacteria;*;g__Bifidobacterium
p__Firmicutes;*;g__[Ruminococcus]
p__Firmicutes;*;g__[Eubacterium]
p__Firmicutes;*;Other
p__Firmicutes;*;g__Dorea
p__Bacteroidetes;*;g__
p__Firmicutes;*;g__Phascolarctobacterium
p__Firmicutes;*;g__Roseburia
p__Firmicutes;*;g__Clostridium
p__Firmicutes;*;g__Ruminococcus
p__Firmicutes;*;g__Coprococcus
p__Firmicutes;*;g__Dialister
p__Firmicutes;*;g__Peptostreptococcus
p__Proteobacteria;*;Other
p__Firmicutes;*;g__Streptococcus
p__Bacteroidetes;*;g__Odoribacter
p__Firmicutes;*;g__Oscillospira
p__Firmicutes;*;g__Anaerostipes
p__Actinobacteria;*;g__Eggerthella
p__Firmicutes;*;g__Enterococcus
Other;*;Other
p__Firmicutes;*;g__Veillonella
p__Firmicutes;*;g__Anaerotruncus
p__Proteobacteria;*;g__Sutterella
p__Bacteroidetes;*;g__Prevotella
p__Bacteroidetes;*;g__Porphyromonas
p__Firmicutes;*;g__Lachnospira
p__Proteobacteria;*;g__Haemophilus
p__Firmicutes;*;g__Turicibacter
p__Firmicutes;*;g__Megasphaera
p__TM7;*;g__
p__Actinobacteria;*;g__Actinomyces
p__Firmicutes;*;g__Holdemania
p__Actinobacteria;*;g__Rothia
Accumulated abundance rank
1
50
Rank Stability Index (in %)
Rank Variability
Differences Variability
RV (50 most abundant)
DV (50 most abundant)
51.join.fna
52.join.fna
53.join.fna
54.join.fna
55.join.fna
Time samples

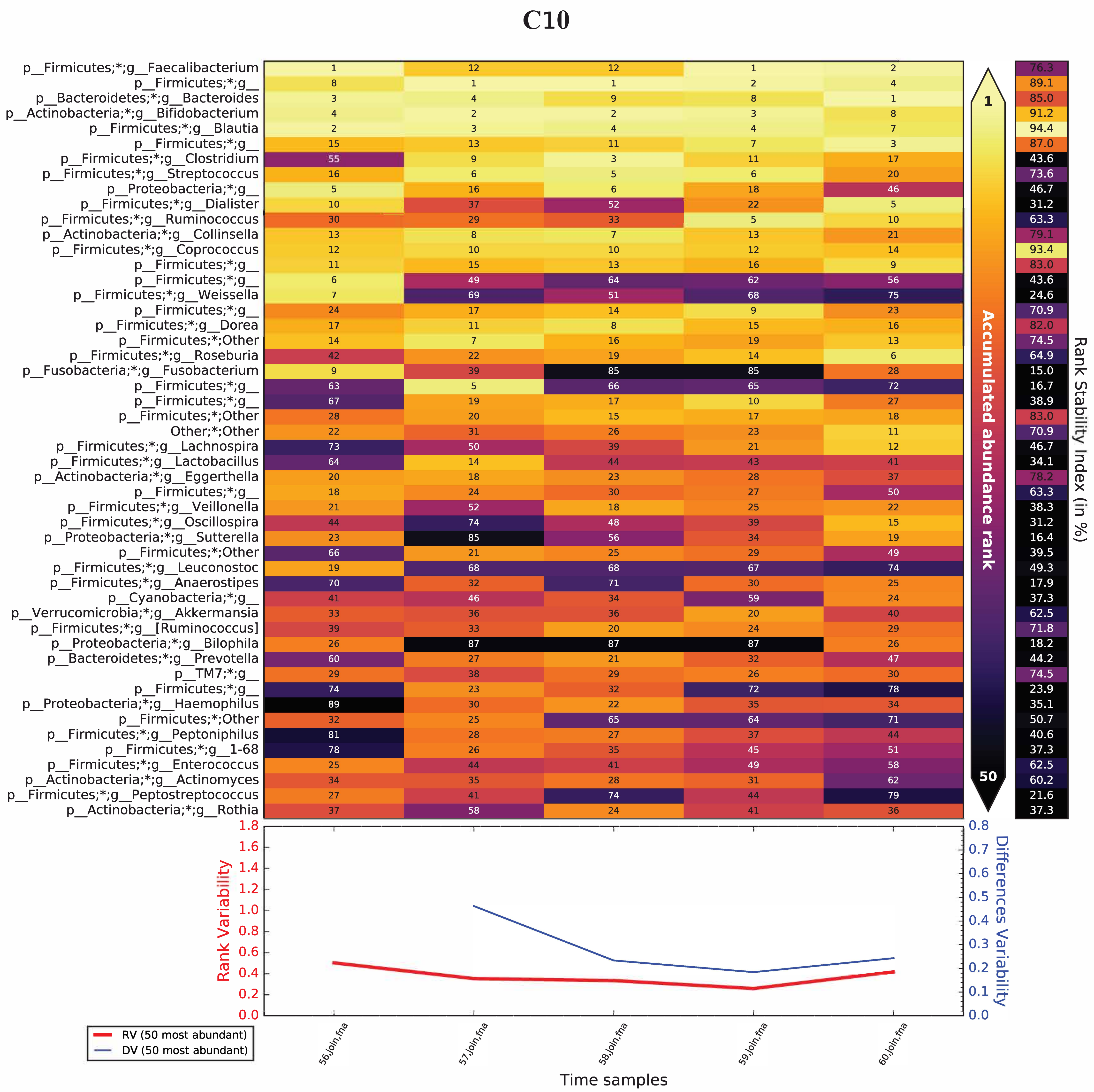
C10
p__Firmicutes;*;g__Faecalibacterium
p__Firmicutes;*;g__
p__Bacteroidetes;*;g__Bacteroides
p__Actinobacteria;*;g__Bifidobacterium
p__Firmicutes;*;g__Blautia
p__Firmicutes;*;g__
p__Firmicutes;*;g__Clostridium
p__Firmicutes;*;g__Streptococcus
p__Proteobacteria;*;g__
p__Firmicutes;*;g__Dialister
p__Firmicutes;*;g__Ruminococcus
p__Actinobacteria;*;g__Collinsella
p__Firmicutes;*;g__Coprococcus
p__Firmicutes;*;g__
p__Firmicutes;*;g__
p__Firmicutes;*;g__Weissella
p__Firmicutes;*;g__
p__Firmicutes;*;g__Dorea
p__Firmicutes;*;Other
p__Firmicutes;*;g__Roseburia
p__Fusobacteria;*;g__Fusobacterium
p__Firmicutes;*;g__
p__Firmicutes;*;g__
p__Firmicutes;*;Other
Other;*;Other
p__Firmicutes;*;g__Lachnospira
p__Firmicutes;*;g__Lactobacillus
p__Actinobacteria;*;g__Eggerthella
p__Firmicutes;*;g__
p__Firmicutes;*;g__Veillonella
p__Firmicutes;*;g__Oscillospira
p__Proteobacteria;*;g__Sutterella
p__Firmicutes;*;Other
p__Firmicutes;*;g__Leuconostoc
p__Firmicutes;*;g__Anaerostipes
p__Cyanobacteria;*;g__
p__Verrucomicrobia;*;g__Akkermansia
p__Firmicutes;*;g__[Ruminococcus]
p__Proteobacteria;*;g__Bilophila
p__Bacteroidetes;*;g__Prevotella
p__TM7;*;g__
p__Firmicutes;*;g__
p__Proteobacteria;*;g__Haemophilus
p__Firmicutes;*;Other
p__Firmicutes;*;g__Peptoniphilus
p__Firmicutes;*;g__1-68
p__Firmicutes;*;g__Enterococcus
p__Actinobacteria;*;g__Actinomyces
p__Firmicutes;*;g__Peptostreptococcus
p__Actinobacteria;*;g__Rothia
1
50
Accumulated abundance rank
Rank Stability Index (in %)
Rank Variability
Differences Variability
RV (50 most abundant)
DV (50 most abundant)
56.join.fna
57.join.fna
58.join.fna
59.join.fna
60.join.fna
Time samples
